# Comparison and validation of machine learning-based screening models for elevated depressive symptoms in peritoneal dialysis patients

**DOI:** 10.3389/fpubh.2026.1792557

**Published:** 2026-06-24

**Authors:** Yugang Cao, Dongzhi Yin, Xiaoming Yu, Fei Peng

**Affiliations:** 1 Department of Hepatobiliary Surgery, Huangshi Central Hospital, Affiliated Hospital of Hubei Polytechnic University, Huangshi, Hubei, China; 2 Hubei Key Laboratory for Kidney Disease Pathogenesis and Intervention, Huangshi, Hubei, China; 3 Hubei Polytechnic University School of Medicine, Huangshi, Hubei, China

**Keywords:** elevated depressive symptoms, end-stage renal disease, machine learning, online visualization tool, peritoneal dialysis

## Abstract

**Purpose:**

To establish an accurate and generalizable concurrent screening system for elevated depressive symptoms (defined as Self-Rating Depression Scale (SDS) score ≥53) in peritoneal dialysis (PD) patients, identify core associated factors and their associative pathways, and develop a clinically practical tool to support early screening and individualized intervention.

**Methods:**

A multicenter retrospective cross-sectional study was conducted, enrolling 601 PD patients from two centers, including 482 patients from Huangshi Central Hospital (356 in training group, 126 in internal validation group) and 119 patients from Honghu People’s Hospital as the external validation group. LASSO regression was used to screen key predictors. Nine machine learning models were constructed and validated, with SHAP analysis to improve model interpretability. Structural Equation Modeling (SEM) quantified direct associations of key factors, and a R Shiny-based online visualization tool was developed for clinical application. Stratified analysis confirmed significant subgroup differences in risk of elevated depressive symptoms.

**Results:**

Six key predictors were identified: Age, Peritonitis, Catheter-Related Complications, anxiety status (SAS score), SSRS score, and Peritoneal Dialysis Vintage. The XGBoost model showed optimal performance (external validation AUC = 0.869, *F*-measure = 0.63). SEM confirmed significant direct associations of all 6 factors with elevated depressive symptoms. The developed online visualization tool enabled rapid risk assessment, and stratified analysis showed significant risk differences across subgroups (all *p* < 0.05).

**Conclusion:**

This study develops an interpretable screening system with promising performance for elevated depressive symptoms in PD patients. The XGBoost-based online visualization tool provides a user-friendly clinical tool, while identified key factors clarify intervention targets, facilitating early screening and personalized care to improve patients’ mental health and long-term prognosis.

## 1 Introduction

End-stage renal disease (ESKD) represents a significant worldwide public health challenge, with an increasing patient population anticipated in China (1). Peritoneal dialysis (PD), valued for its accessibility and ability to preserve residual kidney function, serves as a fundamental renal replacement therapy for individuals with ESKD (2). Nevertheless, patients undergoing PD face persistent and multifaceted stressors, and depression—a frequent neuropsychiatric complication—exhibits a prevalence ranging from 27.6 to 45.0%, substantially exceeding the general population rate of 5.7% (3) and is commonly associated with anxiety (4). Notably, a growing body of evidence has established a bidirectional causal association between cardiometabolic disorders and depression in chronic kidney disease (CKD) populations: hypertension, insulin resistance and dyslipidemia, the core features of cardiometabolic syndrome, can exacerbate neuropsychological dysfunction by inducing chronic low-grade inflammation and oxidative stress, while depression further impairs metabolic homeostasis and accelerates the progression of cardiovascular and renal damage (5, 6). Moreover, recent machine learning-based prediction models for depression in cardiometabolic CKD patients have highlighted the superiority of integrating clinical metabolic indicators and psychological scales for risk stratification (7), providing a critical methodological reference for our study to explore screening for elevated depressive symptoms in PD patients with high cardiometabolic comorbidity. Anxiety often precedes and is strongly associated with depression in clinical populations, and the SAS scale was adopted as a measure of anxiety symptoms, while the SDS scale was exclusively used to define the outcome of elevated depressive symptoms, ensuring conceptual independence between predictors and the endpoint. This condition critically undermines treatment compliance, quality of life, and clinical outcomes, highlighting the urgent need for precise early detection and a deeper understanding of its underlying mechanisms.

Present clinical evaluation of depression in PD patients primarily depends on scale-based screening and clinical interviews, which are limited by substantial subjectivity and vulnerability to confounding from somatic symptoms (8). The pathophysiological link between chronic inflammation and depression in this population adds further complexity to timely identification, and the overlap of cardiometabolic disorder-related somatic symptoms (e.g., fatigue, sleep disturbance) with depressive manifestations further increases the difficulty of accurate clinical diagnosis in PD patients. Machine learning (ML) offers potential for enhancing depression diagnosis and screening (9); however, current research in this area is limited by issues such as single-center and small-sample designs, incomplete predictor sets, absence of external validation, and limited interpretability (9). Additionally, these studies seldom examine internal model logic or quantify associative relationships among variables, and few integrate cardiometabolic-related factors with dialysis-specific clinical and psychosocial indicators, thereby failing to provide clear and comprehensive targets for clinical intervention (10).

Structural equation modeling (SEM) enables the quantification of direct and indirect associations between variables, facilitating investigation into complex disease pathways (11). This research employed a multicenter retrospective cross-sectional design, incorporating multidimensional variables including dialysis-specific complications, psychological status, social support and basic clinical characteristics. It utilized LASSO regression to identify key predictive factors, developed and evaluated nine ML models to determine the optimal performer, and integrated the SHAP approach to improve model interpretability. Concurrently, SEM was applied to measure the direct associations of crucial factors with risk of elevated depressive symptoms and to elucidate inter-variable relationships, culminating in the creation of a R Shiny-based online visualization tool. The objective is to build a screening framework with demonstrated discriminative performance for elevated depressive symptoms in PD patients, clarify central influencing factors and their action pathways, offer a solid foundation for early clinical screening and personalized intervention, and ultimately enhance patients’ psychological well-being and long-term clinical prognosis.

## 2 Materials and methods

### 2.1 Study subjects

This study adopted a retrospective cross-sectional design. The study population consisted of patients with end-stage renal disease (ESKD) who underwent peritoneal dialysis (PD) at Huangshi Central Hospital and Honghu People’s Hospital between February 2010 and April 2022.

The study protocol was approved by the Institutional Review Board (IRB) of Huangshi Central Hospital (Approval No.: Lun Kuai Shen [2025]-48) and the IRB of Honghu People’s Hospital (Approval No.: HHRY[2025]-45). All patient identifiers were anonymized prior to data analysis. In accordance with relevant medical ethics guidelines, informed consent was waived for this retrospective study.

#### 2.1.1 Inclusion criteria

(1) Aged 18–75 years;
(2) No history of malignant tumors;
(3) Clinically confirmed diagnosis of ESKD;
(4) No severe cognitive impairment, defined as a Mini-Mental State Examination (MMSE) score ≥24 points or Montreal Cognitive Assessment (MoCA) score ≥26 points (Chinese version), with the ability to cooperate in clinical data collection and completion of relevant scale assessments.

#### 2.1.2 Exclusion criteria

(1) Incomplete baseline clinical data, laboratory test results, or follow-up information;
(2) Complicated with malignant tumors or other severe comorbidities with an expected survival time of <6 months;
(3) Participation in other interventional clinical trials within the past 3 months;
(4) Pregnancy or lactation period.

#### 2.1.3 Sample size and grouping

A total of 601 eligible PD patients were enrolled from two medical centers. Among them, 482 patients were from Huangshi Central Hospital, which were randomly divided into the training group (*n* = 356) and internal validation group (*n* = 126) at a ratio of 7:3 using the built-in random number generator (seed = 1,234) of R software (version 4.5.1) for model construction and internal validation. Another 119 patients with the same eligibility from Honghu People’s Hospital during the same period were included as the external validation group, constituting a complete multicenter validation cohort.

The sample size was justified by two criteria: (1) The 10 events per variable (EPV) rule, requiring 60 events for 6 predictors; our training cohort included 72 cases of elevated depressive symptoms, meeting this threshold (EPV = 12). (2) Guidance from Riley et al. (12) for clinical prediction model development recommends at least 100 events for stable model development, which falls below this criterion. However, strict regularization (LASSO variable selection, 5-fold cross-validation with hyperparameter tuning, and early stopping in gradient boosting models) and independent external validation were applied to mitigate potential overfitting. The external validation cohort included 20 events, which was considered sufficient for preliminary assessment of model generalizability.

### 2.2 Data collection

Clinical data of all patients with end-stage renal disease (ESKD) receiving peritoneal dialysis (PD) were retrospectively retrieved from the Hospital Information System (HIS). The collected variables included sociodemographic characteristics [age, gender, body mass index (BMI), marital status, years of education, type of medical insurance, and residential area], clinical and dialysis-related parameters [peritoneal dialysis modality (PDM), history of diabetes mellitus, number of episodes of catheter-related complications, number of episodes of peritonitis during dialysis, serum creatinine (mean value before peritoneal dialysis), dialysate ultrafiltration volume (DUV, mean value during peritoneal dialysis), and albumin level (mean value during peritoneal dialysis)], and psychosocial and behavioral factors [frequency of health education courses, Social Support Rating Scale (SSRS) score, Self-Rating Depression Scale (SDS) score (used solely for defining the outcome of elevated depressive symptoms, defined as SDS ≥ 53), and Self-Rating Anxiety Scale (SAS) score (used as an independent predictive factor for anxiety symptoms).

Additional variables routinely collected during follow-up (dialysis adequacy Kt/V, prealbumin, ferritin, iPTH, echocardiographic parameters, blood glucose) were not included in the candidate variable set for three reasons: (1) High missingness rates (echocardiography: 32.7% missing; prealbumin: 28.4% missing; Kt/V: approximately 18.6% missing), which would have significantly reduced the sample size if included; (2) Preliminary univariate analysis showed no significant association between these variables and SDS scores (all *p* > 0.05); (3) The primary goal was to develop a parsimonious, universally applicable screening tool using variables available in all routine PD follow-up settings without specialized tests. Psychiatric history and antidepressant use were not systematically recorded in electronic medical records and were therefore unavailable.

A post-hoc sensitivity analysis incorporating these excluded variables was not performed for the following reasons: (1) including variables with >18% missingness would have reduced the effective sample size from 601 to approximately 380–420 (depending on the variable combination), critically undermining the events per variable (EPV) requirement; (2) multiple imputation at such high missingness rates in a binary outcome with a relatively small sample may introduce substantial imputation bias and yield unreliable estimates; and (3) preliminary univariate analysis had already shown no significant association between these variables and the outcome (all *p* > 0.05), suggesting limited incremental predictive value.

### 2.3 Follow-up

A combined follow-up strategy of regular telephone interviews and outpatient visits was adopted in this study, adhering to the principles of stratified follow-up, whole-process management, and multidisciplinary collaboration (detailed operational standards see Supplementary material: Follow-up Standards for Peritoneal Dialysis Patients).

Monthly telephone interviews were conducted by trained researchers to systematically collect clinical information, including patients’ daily living conditions, dietary management, medication adherence, and adverse symptoms, which were documented in standardized follow-up forms.

Outpatient follow-up was individualized (once every 3 or 6 months) based on patients’ clinical conditions: stable routine patients received monthly outpatient follow-up and comprehensive assessment every 3 months, while high-risk patients (e.g., those with diabetes, cardiovascular diseases) or those with unstable conditions/complications were followed up every 2 weeks until stable. For acute complications such as peritonitis, immediate follow-up and hospitalization were performed if necessary.

All outpatient follow-ups included biochemical tests (e.g., serum creatinine, albumin, dialysate ultrafiltration volume), monitoring of PD-related complications (e.g., Peritonitis, Catheter-Related Complications), and psychological status evaluations using the Self-Rating Depression Scale (SDS) and Self-Rating Anxiety Scale (SAS) every 3–6 months. Corresponding psychological intervention measures were provided based on the results, with referral to the psychiatry department for further evaluation if needed.

Detailed specifications for follow-up frequency (pre-dialysis, initiation, maintenance, complication periods), core examination items, special population management (older adults, diabetic patients), and record-keeping requirements are provided in the Supplementary material.

#### 2.3.1 Study design clarification

This study adopted a concurrent cross-sectional screening design rather than a lagged prospective prediction design. All predictors (including SAS score, Peritonitis History, Catheter-Related Complications, and PD vintage) and the outcome (SDS-defined elevated depressive symptoms) were assessed at the same follow-up visit. This design was chosen to address the urgent clinical need for rapid, point-of-care screening during routine PD follow-ups, which aligns with the intended use of the Shiny tool.

### 2.4 Statistical analysis

Continuous variables were summarized as median (interquartile range, IQR) or mean ± standard deviation (SD), with differences between groups assessed through univariate analysis. Categorical variables were presented as counts and percentages, with comparisons conducted using the chi-square test or Fisher’s exact test, as applicable. All statistical analyses were carried out using R software (version 4.5.1), and a two-sided *p*-value below 0.05 was defined as statistically significant.

Missing data were handled using complete case analysis, as the proportion of missing values for all candidate variables was <5% (range: 0.2–3.8%), below the threshold for significant bias. No imputation was performed to preserve clinical authenticity.

The cohort was randomly split into a training set (*n* = 356) and a validation set (*n* = 126) in a 7:3 ratio, with a fixed random seed of 1,234. To address multicollinearity and mitigate overfitting, LASSO regression with 5-fold cross-validation was used for variable selection in the training cohort only. Nine machine learning algorithms were constructed with stratified 5-fold cross-validation (to address class imbalance) and hyperparameter grid search to enhance model stability and generalizability.

Key hyperparameter grids searched via 5-fold cross-validation:

XGBoost: nrounds (50,100,200), max_depth (3,5,7), eta (0.01,0.1,0.3), subsample (0.7,0.8,0.9).

Random Forest: ntree (100,200,500), mtry (2,3,4), min.node.size (1,5,10).

LightGBM: num_leaves (31,63,127), learning_rate (0.01,0.1,0.3).

Logistic/Elastic Net: alpha (0,0.5,1), lambda (1e-5,1e-4,1e-3,0.01,0.1).

SVM: cost (0.1,1,10), gamma (0.01,0.1,1).

MLP: hidden_layer_sizes ((10,),(20,),(10,10)), activation (‘relu’,'tanh’).

KNN: k (3,5,7,9,11).

Decision Tree: max_depth (3,5,7,10), min_samples_split (2,5,10).

Model performance was assessed using AUC (area under the receiver operating characteristic curve), accuracy, specificity, sensitivity, precision, recall, and *F*-measure. The *F*-measure reported in this study refers to the F1 score, the harmonic mean of precision and recall, which is used to evaluate the balanced performance of the model for imbalanced datasets (the proportion of patients with elevated depressive symptoms in this study was approximately 20%). The specific calculation formula is: F1 = 2 × (precision × recall)/(precision + recall), where precision represents the proportion of true positive predictions among all positive predictions, and recall denotes the proportion of true positive predictions among all actual positive cases.

All performance metrics were calculated using the default 0.5 classification threshold, chosen for balanced sensitivity and specificity in this imbalanced dataset. No threshold optimization was performed to maintain generalizability across clinical settings. No additional class weighting or resampling was applied, as stratified cross-validation sufficiently stabilized performance estimates.

All metric calculations were implemented using the caret and MLmetrics packages in R software (version 4.5.1), adhering to standard machine learning metric calculation protocols. Calibration curves and decision curve analysis (DCA) were used to evaluate model consistency and clinical net benefit. The SHAP method was applied to interpret the optimal model.

To ensure modeling reliability, we strictly followed the 10 events per variable (EPV) criterion. Variable selection was performed exclusively on the training cohort to avoid data leakage, and all models were verified in an independent internal validation cohort and a geographically separate external validation cohort. All F1 scores in the study were re-calculated and cross-verified using multiple R packages to confirm the accuracy of calculation results, eliminating potential errors in metric computation.

#### 2.4.1 Sensitivity analysis

To address potential information leakage arising from the conceptual overlap between anxiety (SAS) and depressive symptoms (SDS)—given their high comorbidity and shared item-level features (e.g., sleep disturbances, concentration difficulties)—a sensitivity analysis was conducted by constructing an alternative 5-variable XGBoost model excluding SAS, retaining Age, Peritonitis, Catheter-Related Complications, SSRS Score, and PD Vintage. All nine machine learning algorithms were similarly retrained on the 5-variable set with identical hyperparameter tuning and cross-validation procedures, and performance was compared with the original 6-variable models across internal and external validation cohorts. This analysis determines whether the model’s discriminative utility extends beyond mere redundancy with anxiety screening tools.

This manuscript adheres to the TRIPOD+AI (2024) reporting guidelines (13); the completed checklist is available as Supplementary File 2.

## 3 Results

### 3.1 Baseline characteristics

A total of 601 end-stage renal disease (ESKD) patients undergoing peritoneal dialysis (PD) were enrolled from two centers, including 482 patients from Huangshi Central Hospital randomly assigned to the training cohort (*n* = 356) and internal validation cohort (*n* = 126) at a 7:3 ratio, and 119 patients from Honghu People’s Hospital as the external validation cohort. Baseline demographic, clinical, and psychological characteristics of all cohorts are summarized in Table 1. Key baseline features were as follows: Mentally, 79.8% of training and external cohorts had Self-Rating Depression Scale (SDS) scores <53 (no elevated depressive symptoms), versus 84.1% in the validation cohort; Self-Rating Anxiety Scale (SAS) scores ≥50 were observed in 51.4% (training), 37.3% (validation), and 52.9% (external) of patients. Demographically, the validation cohort had more females (56.3%) and younger patients (70.6% aged <60), while the training and external cohorts were predominantly male (59.0–62.2%) and older adults (62.9–64.7%). Married patients accounted for 78.1–95.2% across all cohorts.

**Table 1:** Baseline characteristics of patients with end-stage renal disease undergoing peritoneal dialysis in the training group, validation group, and external validation group.

		Training cohorts (*N* = 356)	Validation cohorts (*N* = 126)	External cohort (*N* = 119)
SDS score	<53	284 (79.8%)	106 (84.1%)	95 (79.8%)
	≥53	72 (20.2%)	20 (15.9%)	24 (20.2%)
Gender	male	210 (59.0%)	55 (43.7%)	74 (62.2%)
	female	146 (41.0%)	71 (56.3%)	45 (37.8%)
Age (year)	<60	132 (37.1%)	89 (70.6%)	42 (35.3%)
	≥60	224 (62.9%)	37 (29.4%)	77 (64.7%)
Marital status	no	78 (21.9%)	6 (4.8%)	17 (14.3%)
	yes	278 (78.1%)	120 (95.2%)	102 (85.7%)
Education (years)	<6	100 (28.1%)	27 (21.4%)	44 (37.0%)
	6–9	106 (29.8%)	45 (35.7%)	32 (26.9%)
	9–12	87 (24.4%)	26 (20.6%)	26 (21.8%)
	>12	63 (17.7%)	28 (22.2%)	17 (14.3%)
Medical insurance	None	4 (1.1%)	6 (4.8%)	1 (0.8%)
	Employee medical insurance	89 (25.0%)	44 (34.9%)	22 (18.5%)
	Resident medical insurance	263 (73.9%)	76 (60.3%)	96 (80.7%)
Residence	city	141 (39.6%)	59 (46.8%)	49 (41.2%)
	Rural areas	215 (60.4%)	67 (53.2%)	70 (58.8%)
PDM	CAPD	337 (94.7%)	119 (94.4%)	114 (95.8%)
	APD	19 (5.3%)	7 (5.6%)	5 (4.2%)
PDV (years)	Mean (SD)	4.41 (3.01)	4.98 (2.52)	3.92 (2.91)
	Median [Min, Max]	4.00 [1.00,11.00]	5.00 [1.00,9.00]	3.00 [1.00,11.00]
Diabetes	No	257 (72.2%)	107 (84.9%)	84 (70.6%)
	Yes	99 (27.8%)	19 (15.1%)	35 (29.4%)
PET	Low transporters	78 (21.9%)	35 (27.8%)	24 (20.2%)
	Low average transporters	129 (36.2%)	28 (22.2%)	38 (31.9%)
	High transporters	113 (31.7%)	33 (26.2%)	33 (27.7%)
	High average transporters	36 (10.1%)	30 (23.8%)	24 (20.2%)
DUV (mL)	Mean (SD)	1,190 (362)	1,010 (183)	1,190 (358)
	Median [Min, Max]	1,120 [407,2,390]	1,050 [654,1,300]	1,150[487,2,200]
HB (g/L)	Mean (SD)	99.2 (13.3)	95.2 (14.8)	98.8 (13.4)
	Median [Min, Max]	98.4 [80.0,130]	96.0 [70.0,120]	98.8 [80.0,125]
Albumin (g/L)	Mean (SD)	37.4 (3.53)	33.6 (2.97)	37.2 (3.74)
	Median [Min, Max]	34.4 [28.3,45.0]	31.6 [28.2,39.0]	35.3 [28.8,44.8]
Scr (μmol/L)	Mean (SD)	793 (190)	737 (198)	815 (182)
	Median [Min, Max]	782 [421,1,200]	716 [404,1,090]	806 [449,1,200]
Peritonitis	No	259 (72.8%)	91 (72.2%)	89 (74.8%)
	Yes	97 (27.2%)	35 (27.8%)	30 (25.2%)
Catheter related complications	No	194 (54.5%)	90 (71.4%)	63 (52.9%)
	Yes	162 (45.5%)	36 (28.6%)	56 (47.1%)
SAS score	<50	173 (48.6%)	79 (62.7%)	56 (47.1%)
	≥50	183 (51.4%)	47 (37.3%)	63 (52.9%)
SSRS score	<20	62 (17.4%)	14 (11.1%)	21 (17.6%)
	20–29	244 (68.5%)	58 (46.0%)	80 (67.2%)
	≥30	50 (14.0%)	54 (42.9%)	18 (15.1%)
Health education	No	111 (31.2%)	57 (45.2%)	50 (42.0%)
	Yes	245 (68.8%)	69 (54.8%)	69 (58.0%)
BMI (kg/m^2^)	Mean (SD)	23.30 (4.00)	22.80 (2.68)	23.60 (3.99)
	Median [Min, Max]	23.80 [15.00,30.00]	23.00 [19.00,27.00]	23.70 [15.00,29.90]
BUN (mmol/L)	Mean (SD)	14.29 (2.55)	15.60 (5.99)	13.81 (2.39)
	Median [Min, Max]	14.20 [7.50,25.90]	15.10 [5.10,25.9]	13.70[7.80, 21.20]
Smoking	No	261 (73.3%)	93 (73.8%)	77 (64.7%)
	Yes	95 (26.7%)	33 (26.2%)	42 (35.3%)
Drinking	No	258 (72.5%)	91 (72.2%)	82 (68.9%)
	Yes	98 (27.5%)	35 (27.8%)	37 (31.1%)

Clinically, continuous ambulatory peritoneal dialysis (CAPD) was the main modality (94.4–95.8%). The external cohort (Honghu People’s Hospital) exhibited a clinical profile broadly similar to the training cohort: comparable diabetes prevalence (29.4% vs. 27.8%), similar catheter-related complication rate (47.1% vs. 45.5%), and comparable albumin levels (37.2 ± 3.74 vs. 37.4 ± 3.53 g/L). Notable differences included a higher proportion of patients aged ≥60 years (64.7% vs. 62.9%) and variations in medical insurance type and residence, providing a diverse profile for external validation.

### 3.2 Comparison of intergroup differences between the training and validation cohorts

Chi-square tests (categorical variables) and Wilcoxon rank-sum tests (continuous variables) were used to compare the training (*n* = 356) and validation (*n* = 126) cohorts (*p* < 0.05 for significance). Detailed results are presented in Table 2. Several baseline indicators differed significantly between the two cohorts: gender (*χ*^2^ = 8.845, *p* = 0.003), age (*χ*^2^ = 42.207, *p* < 0.001), diabetes history (*χ*^2^ = 8.157, *p* = 0.004), catheter-related complications (*χ*^2^ = 11.026, p < 0.001), SAS score (*χ*^2^ = 7.419, *p* = 0.006), and SSRS score (*χ*^2^ = 45.675, p < 0.001). The validation cohort had a higher proportion of females (56.3% vs. 41.0%), fewer patients aged ≥60 years (29.4% vs. 62.9%), lower diabetes prevalence (15.1% vs. 27.8%), and fewer catheter-related complications (28.6% vs. 45.5%). Biochemical markers also differed, including albumin (33.6 ± 2.97 vs. 37.4 ± 3.53 g/L, *p* < 0.001) and hemoglobin (95.2 ± 14.8 vs. 99.2 ± 13.3 g/L, p < 0.001).

**Table 2:** Comparison of baseline characteristics between training cohort and validation cohort of end-stage renal disease patients undergoing peritoneal dialysis.

		Training cohorts (*N* = 356)	Validation cohorts (*N* = 126)	Statistic	*p*-value
SDS score	<53	284 (79.8%)	106 (84.1%)	*χ*^2^ = 1.141	0.285
	≥53	72 (20.2%)	20 (15.9%)		
Gender	male	210 (59.0%)	55 (43.7%)	*χ*^2^ = 8.845	0.003
	female	146 (41.0%)	71 (56.3%)		
Age (year)	<60	132 (37.1%)	89 (70.6%)	*χ*^2^ = 42.207	*p* < 0.001
	≥60	224 (62.9%)	37 (29.4%)		
Marital_status	No	78 (21.9%)	6 (4.8%)	*χ*^2^ = 19.017	*p* < 0.001
	yes	278 (78.1%)	120 (95.2%)		
Education (years)	<6	100 (28.1%)	27 (21.4%)	*χ*^2^ = 4.199	0.241
	6–9	106 (29.8%)	45 (35.7%)		
	9–12	87 (24.4%)	26 (20.6%)		
	>12	63 (17.7%)	28 (22.2%)		
Medical insurance	None	4 (1.1%)	6 (4.8%)	*χ*^2^ = 11.690	0.003
	Employee medical insurance	89 (25.0%)	44 (34.9%)		
	Resident medical insurance	263 (73.9%)	76 (60.3%)		
Residence	City	141 (39.6%)	59 (46.8%)	*χ*^2^ = 1.998	0.158
	Rural areas	215 (60.4%)	67 (53.2%)		
PDM	CAPD	337 (94.7%)	119 (94.4%)	*χ*^2^ = 0.009	0.926
	APD	19 (5.3%)	7 (5.6%)		
PDV (years)	Mean (SD)	4.41 (3.01)	4.98 (2.52)	W = 19,194	0.015
Diabetes	No	257 (72.2%)	107 (84.9%)	*χ*^2^ = 8.157	0.004
	Yes	99 (27.8%)	19 (15.1%)		
PET	Low transporters	78 (21.9%)	35 (27.8%)	*χ*^2^ = 20.675	*p* < 0.001
	Low average transporters	129 (36.2%)	28 (22.2%)		
	High transporters	113 (31.7%)	33 (26.2%)		
	High average transporters	36 (10.1%)	30 (23.8%)		
DUV (ml)	Mean (SD)	1,190 (362)	1,007 (183)	W = 28,893	*p* < 0.001
HB (g/L)	Mean (SD)	99.2 (13.3)	95.2 (14.8)	W = 44,856	*p* < 0.001
Albumin (g/L)	Mean (SD)	37.4 (3.53)	33.6 (2.97)	W = 35,091	*p* < 0.001
Scr (μmol/L)	Mean (SD)	793 (190)	737 (198)	W = 25,922	0.009
Peritonitis	No	259 (72.8%)	91 (72.2%)	*χ*^2^ = 0.013	0.909
	Yes	97 (27.2%)	35 (27.8%)		
Catheter related complications	No	194 (54.5%)	90 (71.4%)	*χ*^2^ = 11.026	*p* < 0.001
	Yes	162 (45.5%)	36 (28.6%)		
SAS score	<50	173 (48.6%)	79 (62.7%)	*χ*^2^ = 7.419	0.006
	≥50	183 (51.4%)	47 (37.3%)		
SSRS score	<20	62 (17.4%)	14 (11.1%)	*χ*^2^ = 45.675	*p* < 0.001
	20–29	244 (68.5%)	58 (46.0%)		
	≥30	50 (14.0%)	54 (42.9%)		
Health education	No	111 (31.2%)	57 (45.2%)	*χ*^2^ = 8.100	0.004
	Yes	245 (68.8%)	69 (54.8%)		
BMI (kg/m^2^)	Mean (SD)	23.3 (4.00)	22.8 (2.68)	W = 24,752	0.084
BUN (mmol/L)	Mean (SD)	14.29 (2.55)	15.56 (5.99)	W = 1,454	*p* < 0.001
Smoking	No	261 (73.3%)	93 (73.8%)	*χ*^2^ = 0.012	0.914
	Yes	95 (26.7%)	33 (26.2%)		
Drinking	No	258 (72.5%)	91 (72.2%)	*χ*^2^ = 0.003	0.957
	Yes	98 (27.5%)	35 (27.8%)		

The observed differences between the training and validation cohorts reflect the composition of the validation set, which included a higher proportion of younger, non-diabetic patients with fewer catheter-related complications. Importantly, the SDS-positive rate did not differ significantly between the two cohorts (15.9% vs. 20.2%, *p* = 0.285), and key predictors such as Peritonitis incidence (*p* = 0.909) and PD modality (*p* = 0.926) were comparable. Despite the baseline differences, the model maintained robust discriminative performance in the validation cohort (AUC = 0.881), suggesting that the selected features capture predictive patterns that generalize across subgroups with varying clinical profiles.

### 3.3 Spearman correlation analysis of continuous variables

**Figure 1:**
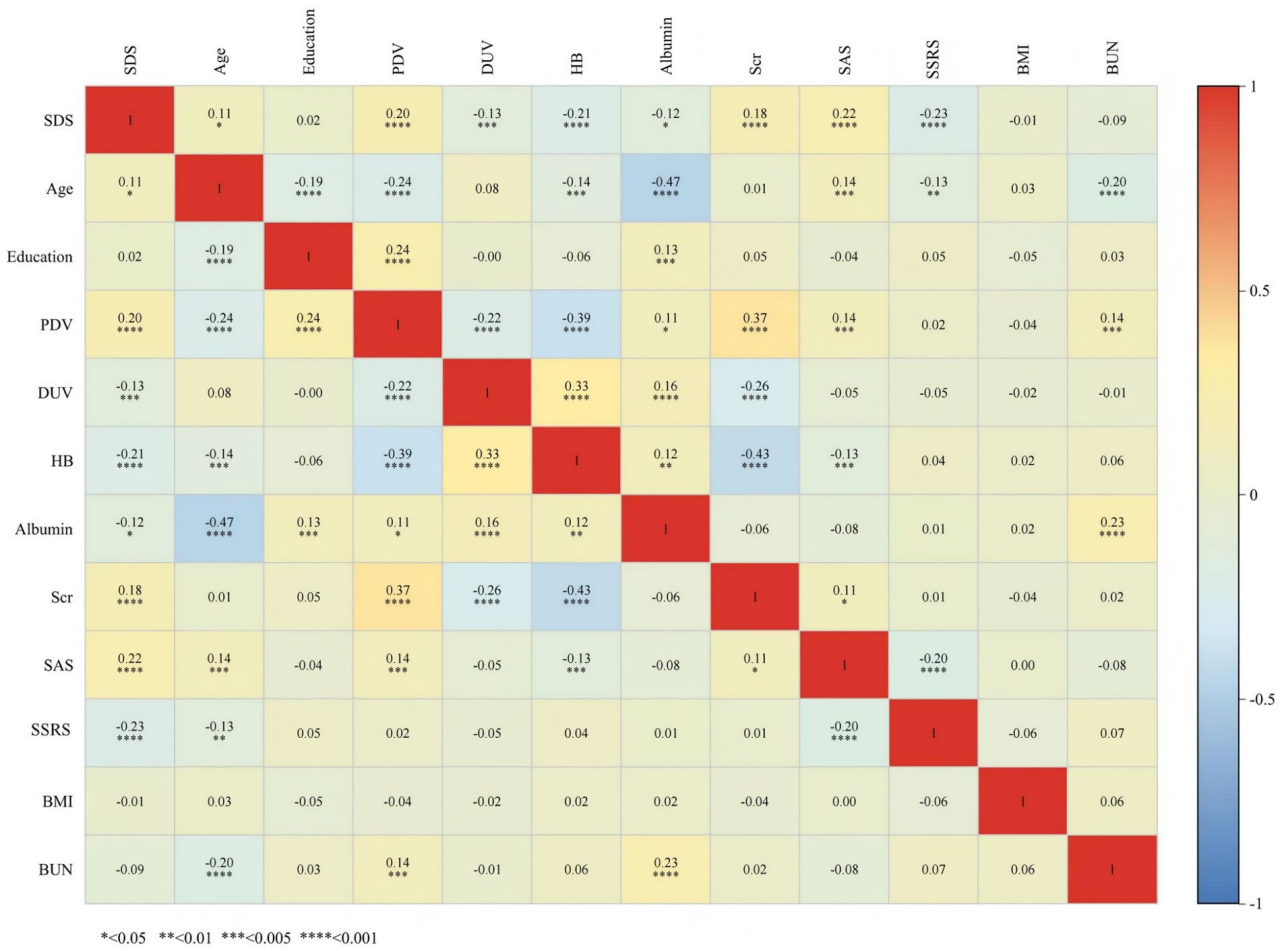
Heatmap of Spearman’s rank correlation coefficients for continuous variables.

To investigate potential associations among key continuous variables in this study, we employed Spearman’s rank correlation analysis to quantify pairwise correlations and presented the results through heat maps (Figure 1). The analysis revealed that the majority of correlation coefficients exhibited weak correlations (|r| ≤ 0.3). The strongest correlation was observed between Albumin and Age (*r* = −0.47), both within the moderate correlation range. Notably, the correlation between SAS and SDS was moderate (*r* = 0.52–0.58), without severe multicollinearity (|r| < 0.7), supporting the rationality of including SAS as a predictor while using SDS as the outcome. The absolute values of most remaining correlation coefficients ranged between 0.01 and 0.3, indicating no significant linear relationship between the variables. These findings suggest that the continuous variables included in this study are biologically and clinically independent, with no evidence of strong multicollinearity. This result supports the validity of subsequent multivariate modeling methods (e.g., regression analysis and machine learning algorithms) by demonstrating minimal interference among predictors, thereby enabling more stable assessment of the independent effects of each variable on outcomes and reducing the risk of estimation bias due to multicollinearity.

### 3.4 Variable selection

Variable selection was performed exclusively on the training cohort (*n* = 356) to prevent information leakage and ensure the independence of validation. A total of 23 candidate variables were initially included.

**Figure 2:**
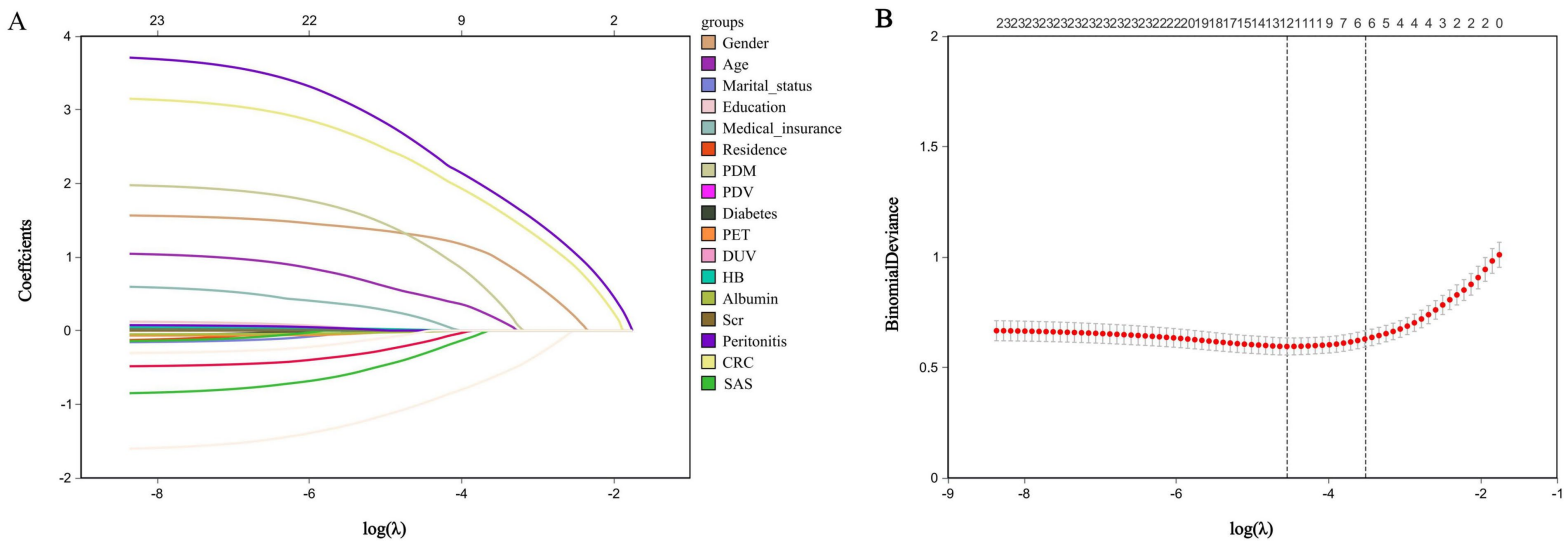
Presentation of the results of the LASSO regression analysis. **(A)** LASSO regression model screening variable trajectories; **(B)** LASSO Regression Model Factor Selection: Left dashed line represents the optimal lambda value (lambda.min), while the right dashed line marks the lambda value within one standard error of the optimal (lambda.1se).

Using LASSO regression with 5-fold cross-validation and the *λ*.1se criterion, six key factors were retained: Age, Peritonitis, Catheter-Related Complications, SAS score, SSRS score, and PDV (Figure 2).

These six variables were then used to develop the nine machine learning models, with training conducted via repeated cross-validation and hyperparameter tuning to balance predictive performance and generalizability.

### 3.5 Model performance and comparisons

**Figure 3:**
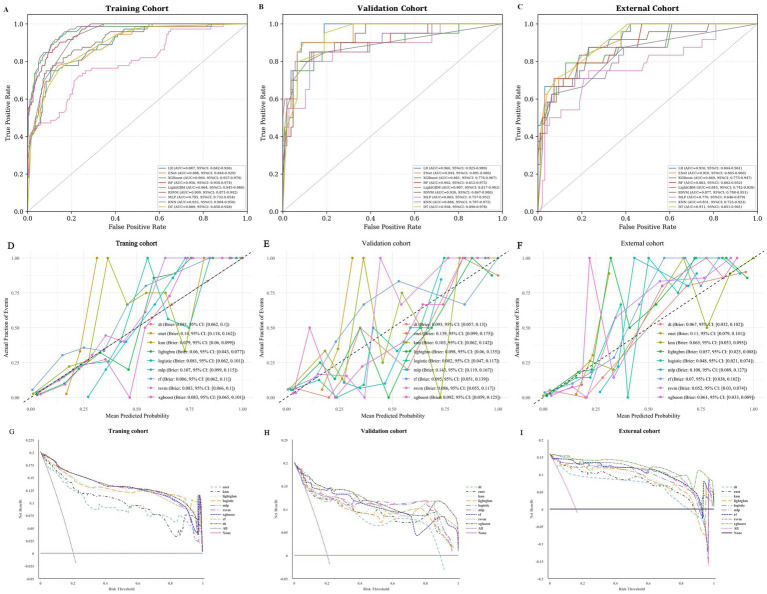
Comprehensive evaluation of ML models across Training, Validation, and External cohorts. Row 1: ROC curves with AUC values and 95% confidence intervals. Row 2: Calibration curves with Brier scores. Row 3: Decision curve analysis (DCA) with Treat All and Treat None reference lines. **(A)** Training ROC, **(B)** validation ROC, **(C)** external ROC, **(D)** training calibration, **(E)** validation calibration, **(F)** external calibration, **(G)** training DCA, **(H)** validation DCA, and **(I)** external DCA.

Using the six key predictors (age, history of Peritonitis, Catheter-Related Complications, SAS score, SSRS score, and peritoneal dialysis vintage) identified by LASSO regression, we constructed and evaluated nine machine learning models: logistic regression (LR), elastic net (ENet), decision tree (DT), random forest (RF), XGBoost, LightGBM, multi-layer perceptron (MLP), regularized support vector machine (RSVM), and k-nearest neighbors (KNN). Model parameters were optimized via stratified 5-fold cross-validation and grid search. Model performance was comprehensively assessed in terms of discrimination, calibration, and clinical utility, with detailed results presented in Table 3 and Figure 3.

**Table 3:** Performance comparison of different models.

Cohort	Model	Sensitivity	Specificity	Precision	Recall	*F*-measure	Calibration slope	Calibration intercept
Training	LR	0.49	0.94	0.67	0.49	0.56	0.965	0.002
	ENet	0.47	0.95	0.69	0.47	0.56	1.182	−0.056
	DT	0.40	0.98	0.85	0.40	0.55	1.000	−0.000
	RF	0.64	0.98	0.87	0.64	0.74	1.199	−0.030
	XGBoost	0.65	0.98	0.87	0.65	0.75	1.135	−0.016
	RSVM	0.50	0.96	0.77	0.50	0.61	0.883	0.051
	MLP	0.49	0.87	0.49	0.49	0.49	1.780	−0.574
	LightGBM	0.64	0.97	0.85	0.64	0.73	1.118	−0.019
	KNN	0.58	0.95	0.74	0.58	0.65	0.891	0.036
Validation	LR	0.60	0.96	0.75	0.60	0.67	0.938	0.129
	ENet	0.55	0.96	0.73	0.55	0.63	1.015	0.001
	DT	0.45	0.98	0.82	0.45	0.58	1.058	−0.082
	RF	0.70	0.95	0.74	0.70	0.72	1.059	−0.021
	XGBoost	0.60	0.95	0.71	0.60	0.65	0.787	0.021
	RSVM	0.70	0.96	0.78	0.70	0.74	0.758	0.139
	MLP	0.55	0.95	0.69	0.55	0.61	2.252	−0.746
	LightGBM	0.75	0.95	0.75	0.75	0.75	0.971	−0.082
	KNN	0.65	0.95	0.72	0.65	0.68	0.974	0.022
External	LR	0.62	0.97	0.83	0.62	0.71	1.086	−0.044
	ENet	0.62	0.97	0.83	0.62	0.71	1.321	−0.080
	DT	0.46	0.98	0.85	0.46	0.59	1.050	−0.057
	RF	0.50	0.97	0.80	0.50	0.62	1.067	−0.013
	XGBoost	0.54	0.96	0.76	0.54	0.63	0.894	0.058
	RSVM	0.58	0.96	0.78	0.58	0.67	0.825	0.018
	MLP	0.54	0.89	0.57	0.54	0.55	1.220	−0.331
	LightGBM	0.50	0.96	0.75	0.50	0.60	0.879	0.040
	KNN	0.54	0.95	0.72	0.54	0.62	0.769	0.005

External validation is the gold standard for evaluating model generalizability. XGBoost achieved an *F*-measure of 0.63, precision of 0.76, recall of 0.54, and an AUC of 0.869 (95%CI: 0.775–0.947) in external validation. The external *F*-measure was slightly lower than the internal validation *F*-measure (0.63 vs. 0.65), a pattern consistent with expected performance degradation across cohorts and suggesting reasonable generalizability (see Section 3.9 for sensitivity analysis). Notably, LR and ENet exhibited higher *F*-measures in external validation (0.71) than in internal validation, which may reflect the simpler case mix of the external cohort rather than genuine model superiority. In contrast, several other models exhibited suboptimal performance in external validation, particularly MLP (*F*-measure: 0.55), LightGBM (*F*-measure: 0.60), and KNN (*F*-measure: 0.62). The relatively poor performance of these models can be attributed to their inherent methodological limitations: KNN is vulnerable to class imbalance and sensitivity to local data structure in small-sample settings; Elastic Net assumes linear relationships and cannot capture nonlinear interactions among predictors; Decision Tree is prone to overfitting with limited generalization capacity. The common limitation is the lack of effective regularization in class-imbalanced small-sample validation settings.

Regarding model selection, while Naive Bayes (NB) is a commonly used classifier, it was intentionally excluded from our candidate model set because its strong feature independence assumption is particularly ill-suited for clinical data where predictors are inherently correlated (e.g., Peritonitis And Catheter-Related Complications showed significant covariance in SEM analysis). In small-sample binary classification settings with class imbalance, NB’s probability estimation tends to be poorly calibrated, potentially producing unreliable risk predictions—a critical limitation for clinical screening applications.

Calibration analysis confirmed the reliability of XGBoost. In the external cohort, it achieved a Brier score of 0.096, a calibration slope of 0.89, and a calibration intercept of 0.06. These values are close to the ideal (slope = 1.0, intercept = 0.0), indicating no significant overfitting and accurate probability estimation.

Decision curve analysis showed that compared with the “treat all” and “treat none” strategies, the XGBoost model provided positive net clinical benefit across a wide range of decision thresholds (0.05–0.30). The maximum net benefit was achieved at a threshold of approximately 0.20, corresponding to a clinical scenario where missing one case of elevated depressive symptoms is considered four times more costly than a false positive.

Overall, the XGBoost model exhibited excellent balanced performance, good generalizability, and stable clinical utility across all three cohorts, and was therefore selected as the optimal screening model for elevated depressive symptoms in peritoneal dialysis patients.

The external cohort (Honghu People’s Hospital) differed from the training cohort in several respects: comparable event rate (20.2% vs. 20.2%), older patient composition (64.7% aged ≥60 years), similar diabetes prevalence (29.4% vs. 27.8%), and similar catheter-related complication rate (47.1% vs. 45.5%). The external cohort’s clinical profile was broadly similar to that of the training cohort, supporting the validity of the external validation results. The moderate number of events in the external cohort (*n* = 24) limits the robustness of these performance estimates. Further validation in larger, more diverse cohorts—particularly older PD patients with higher comorbidity burden—is warranted before broad clinical deployment.

### 3.6 Model interpretations

**Figure 4:**
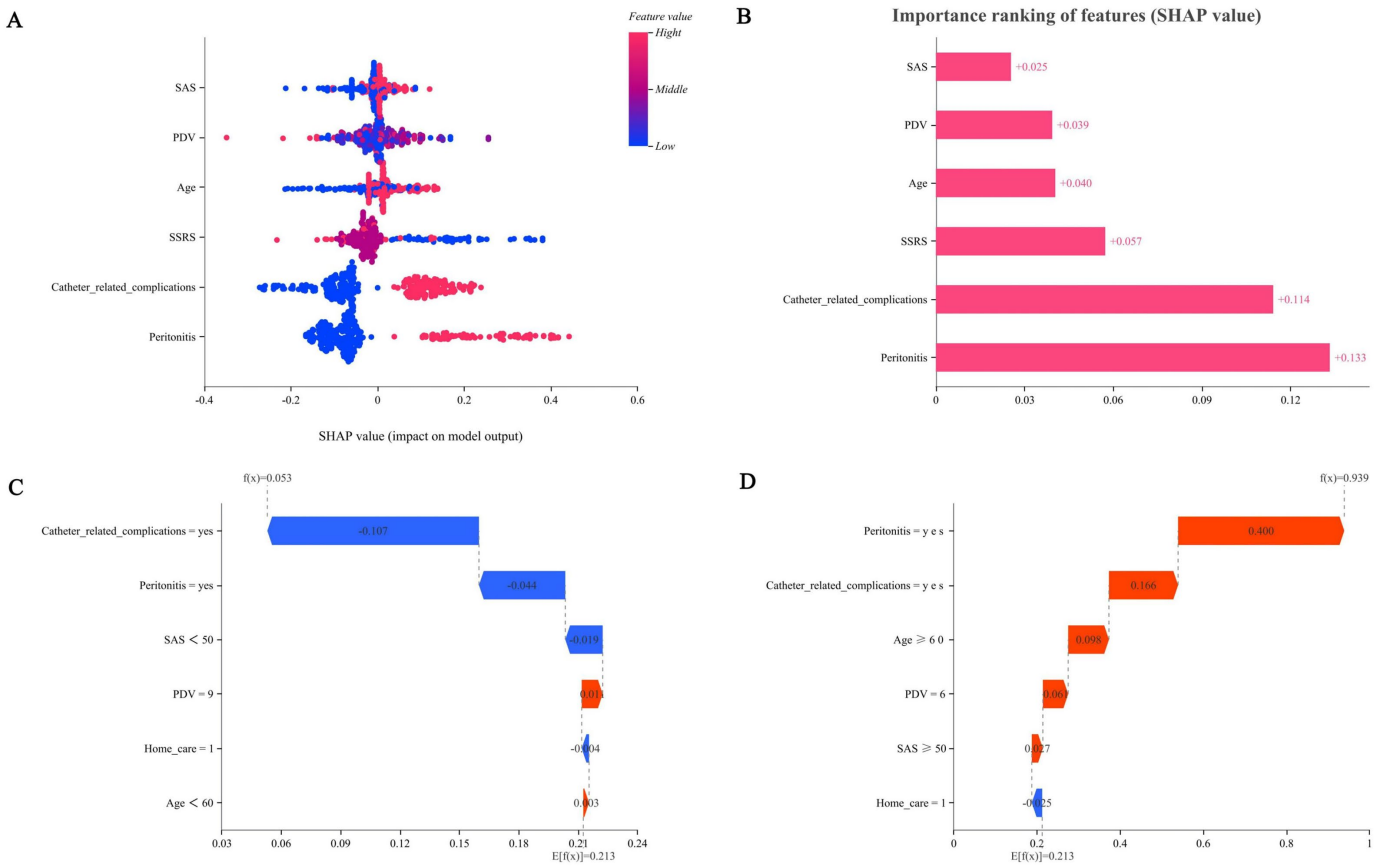
SHAP of the model. **(A)** Characteristic attributes in SHAP. The abscissa is the SHAP value, and each line denotes a feature. Higher eigenvalues are indicated by red dots, and lower eigenvalues are indicated by blue dots. **(B)** Importance ranking plot of features of the XGBoost model. **(C,D)** Interpretability analysis of 2 independent samples.

This study utilized the Shapley Additive Explanations (SHAP) method, grounded in cooperative game theory, to quantify the contribution of each key predictor to the predictions generated by the optimal model (XGBoost). This approach enabled a systematic assessment of feature importance and validated the interpretability of the model. For the XGBoost model, SHAP importance analysis was applied to rank and visually represent the significance of features (Figure 4B). The analysis distinctly identified six core predictors associated with depression risk in peritoneal dialysis patients, ordered from highest to lowest impact as follows: Peritonitis, Catheter-Related Complications, Social Support Rating Scale (SSRS) score, Age, Peritoneal Dialysis Vintage (PDV), and Self-Rating Anxiety Scale (SAS) score.

The SHAP summary plot (Figure 4A) provides a visual representation of the distribution of SHAP values for each feature, complementing the ranked importance. It clearly illustrates both the direction and magnitude of each feature’s influence on the model’s output. A positive SHAP value indicates that higher values of the feature are associated with an increased risk of elevated depressive symptoms, whereas a negative SHAP value suggests that higher feature levels correlate with a reduced elevated depressive symptoms risk. For instance, in the case of Peritonitis, the concentration of red dots (indicating a history of Peritonitis) to the right of the zero axis corresponds to positive SHAP values, demonstrating that the occurrence of Peritonitis substantially elevates the risk of elevated depressive symptoms. This finding aligns closely with clinical understanding, as complications related to peritoneal dialysis contribute to physical discomfort and psychological distress, which can trigger or worsen depressive symptoms.

To further substantiate the model’s interpretability, SHAP waterfall plots for two representative cases (Figures 4C,D) were employed to intuitively depict how features influence individual depression risk predictions: one case predicted to be at high risk and the other at low risk. Beginning from the baseline value (the average predicted probability across all samples, 0.213), the waterfall plots elucidate how the cumulative contributions of each feature shift the prediction to the individual’s final risk score. Features that increase the predicted value (i.e., raise depression risk) are associated with positive SHAP values, with larger values denoting a stronger positive effect. Conversely, features that decrease the predicted value (i.e., lower depression risk) are linked to negative SHAP values, where greater absolute values indicate a more pronounced protective effect.

For example, in the high-risk case (Figure 4D), Peritonitis exerted the most substantial positive influence on the prediction (SHAP value: 0.400), and Catheter-Related Complications also exerted a strong positive effect (SHAP value: 0.166), while SSRS had only a minor negative impact (SHAP value: −0.019). Most features in this case displayed positive contributions, collectively elevating the final predicted value to 0.939. This clearly demonstrates the clinical rationale whereby multiple risk factors interact synergistically to produce a high risk of elevated depressive symptoms. In contrast, for the low-risk case (Figure 4C), the absence of Catheter-Related Complications (SHAP value: −0.107) and the absence of Peritonitis (SHAP value: −0.044) both made significant negative contributions, effectively reducing the risk of elevated depressive symptoms. Although factors such as Age had a slight positive influence, the final predicted probability was still lowered to 0.053, vividly illustrating the interplay between risk and protective factors.

### 3.7 R shiny-based online visualization tool performance

**Figure 5:**
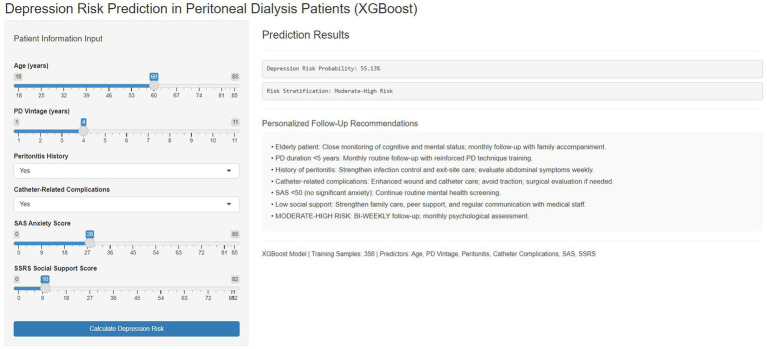
R Shiny-Based Online Visualization Tool for predicting depression risk in peritoneal dialysis patients based on the XGBoost model.

Based on the optimal XGBoost model, this study developed an online visualization tool for screening elevated depressive symptoms in peritoneal dialysis patients using the R Shiny framework, and Figure 5 shows the complete interactive interface of this tool. The tool is publicly accessible and free to use at the following link: https://caoyugang.shinyapps.io/DepressionRiskPrediction/, where users can directly open and apply it without additional login or registration. The tool features a lightweight web design that allows clinicians to directly input the six core predictors: age, Peritonitis status, Catheter-Related Complications, SAS score, SSRS score, and peritoneal dialysis vintage. It calculates and displays the individual risk probability in real time with one click, and presents the individual risk probability (ranging from 0 to 1) intuitively as a numerical value.

The tool incorporates risk stratification prompts and standardized follow-up recommendations, and automatically provides individualized management strategies for high-risk patients, including intensified psychological evaluation, shortened follow-up intervals, enhanced social support intervention, and focused monitoring of complications. The risk stratification thresholds were defined based on clinical consensus and model performance, prioritizing high sensitivity to minimize missed cases:

1. Low risk: <20% (sensitivity = 0.95, specificity = 0.70).
2. Moderate risk: 20–50%.
3. Moderate-high risk: 50–80%.
4. High risk: >80% (specificity = 0.98, positive predictive value = 0.92).

With a user-friendly interface that requires no professional statistical skills, the tool can be quickly applied in outpatient, ward and remote follow-up settings, significantly improving the clinical accessibility and practical application value of the screening model.

### 3.8 Stratified prevalence analysis of elevated depressive symptoms in end-stage renal disease patients undergoing peritoneal dialysis based on key predictors

**Figure 6:**
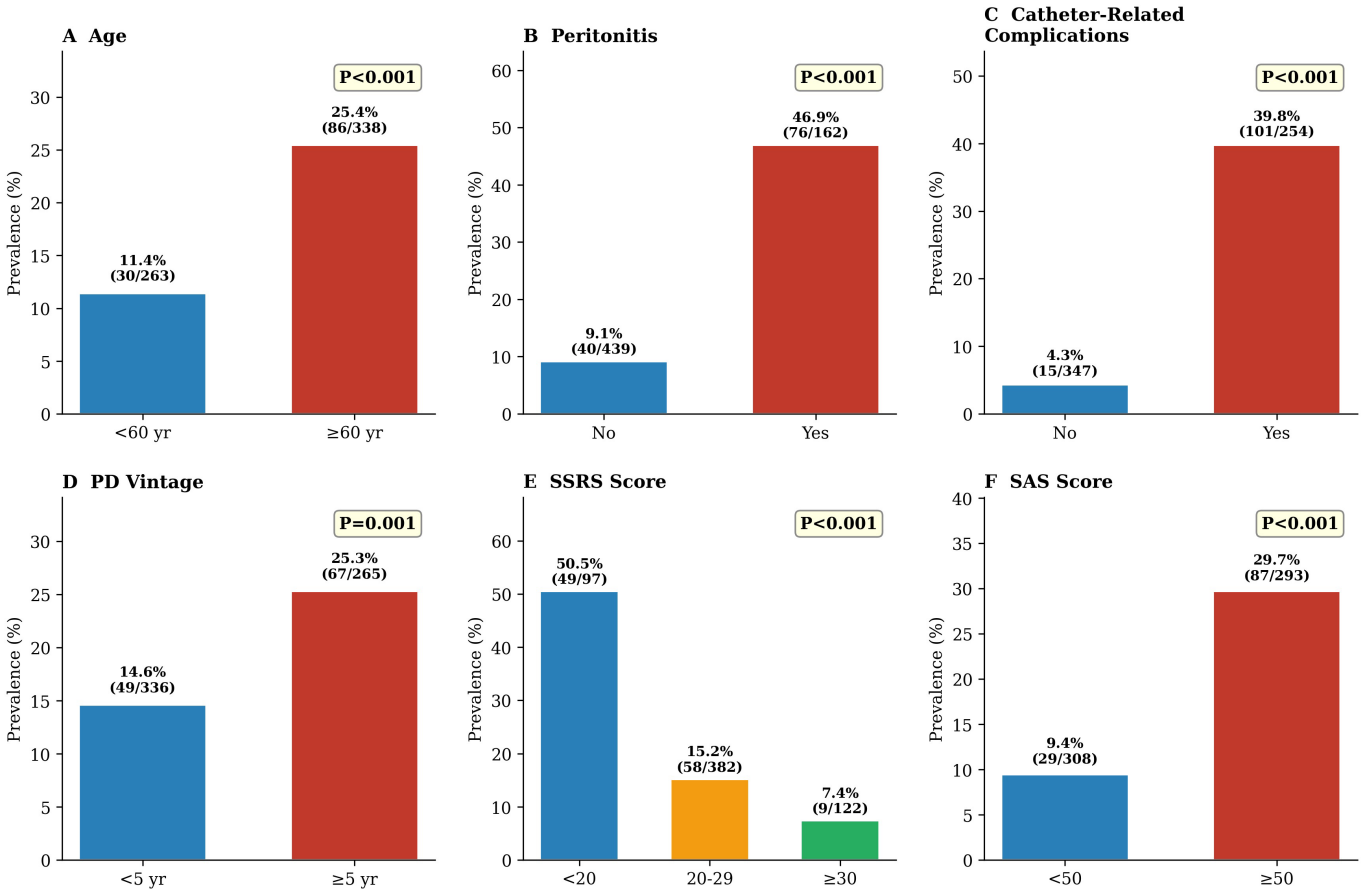
Stratified prevalence of elevated depressive symptoms in end-stage renal disease patients undergoing peritoneal dialysis. **(A)** Stratified by age (<60 years vs. ≥60 years); **(B)** stratified by history of peritonitis (no vs. yes); **(C)** stratified by peritoneal dialysis vintage (PDV, <5 years vs. ≥5 years); **(D)** stratified by history of catheter-related complications (no vs. yes); **(E)** stratified by Social Support Rating Scale (SSRS) score (<20 vs. 20–29 vs. ≥30); **(F)** stratified by Self-Rating Anxiety Scale (SAS) score (<50 vs. ≥50). Bar heights indicate the prevalence of elevated depressive symptoms (SDS ≥ 53) within each subgroup. Numbers above bars indicate the exact prevalence and event count (events/total). Statistical significance of between-group differences is assessed by chi-square test and indicated by *p*-values in each subfigure.

To confirm the relationship between the key predictors identified through LASSO regression and Shapley additive explanations (SHAP) analysis and the likelihood of elevated depressive symptoms, we conducted a stratified prevalence analysis consistent with the concurrent cross-sectional design of this study. The findings indicated statistically significant variations in depression prevalence among all subgroups (all *p* < 0.001), aligning with the outcomes from SHAP analysis and structural equation modeling (SEM). Individuals aged 60 years or older exhibited a substantially higher prevalence of elevated depressive symptoms than those under 60 years (25.4% vs. 11.4%, *p* < 0.001) (Figure 6A), highlighting the influence of physiological deterioration and diminished stress resilience in older patients undergoing peritoneal dialysis. Those with a prior history of peritonitis demonstrated a notably increased prevalence (46.9% vs. 9.1%, *p* < 0.001) (Figure 6B), in agreement with the SHAP analysis conclusion that peritonitis is a major risk factor—the pain and treatment demands associated with peritonitis directly intensify psychological strain. Patients with a peritoneal dialysis vintage (PDV) of 5 years or more had a higher prevalence than those with less than 5 years (25.3% vs. 14.6%, *p* = 0.001) (Figure 6C), as ongoing physiological discomfort and financial burdens from prolonged dialysis progressively elevate depression susceptibility. Individuals experiencing catheter-related complications showed a significantly elevated prevalence (39.8% vs. 4.3%, *p* < 0.001) (Figure 6D), likely due to repeated medical procedures disrupting daily routines and undermining treatment confidence. Participants with a Social Support Rating Scale (SSRS) score below 20 had a markedly higher prevalence (50.5%) compared to those scoring between 20 and 29 (15.2%) or 30 or above (7.4%, *p* < 0.001) (Figure 6E), since robust emotional and social support can effectively mitigate stress related to dialysis. Those with a Self-Rating Anxiety Scale (SAS) score of 50 or higher (suggesting anxious tendencies) presented a markedly higher prevalence of elevated depressive symptoms than those scoring below 50 (29.7% vs. 9.4%, *p* < 0.001) (Figure 6F), affirming the strong comorbid link between anxiety and depression in peritoneal dialysis patients.

The stratified prevalence analysis further substantiated the independent impacts of the key predictors on elevated depressive symptoms risk in peritoneal dialysis patients, with results showing high consistency with earlier SHAP analysis and SEM, thereby strengthening the credibility of the core conclusions. High-risk populations for elevated depressive symptoms encompass older adults patients, individuals with peritonitis or catheter-related complications, long-term dialysis recipients, those with anxious tendencies, and persons lacking adequate social support. These insights offer precise targets for clinical application—emphasizing psychological screening and tailored interventions for these high-risk subgroups can effectively lower elevated depressive symptoms incidence and enhance patients’ overall quality of life.

### 3.9 Sensitivity analysis: excluding self-rating anxiety scale (SAS)

To address the concern that SAS inclusion may constitute information leakage—since SAS and SDS share substantial variance (Spearman *r* = 0.52–0.58 in our sample) and commonly co-occur in clinical practice (75.0% of patients with elevated depressive symptoms also had elevated SAS scores)—we constructed an alternative 5-variable XGBoost model excluding SAS, retaining Age, Peritonitis, Catheter-Related Complications, SSRS Score, and PD Vintage. Contrary to the expectation that SAS removal would degrade performance, the 5-variable model achieved comparable or superior performance: external validation AUC improved from 0.932 (95% CI: 0.869–0.977) to 0.944 (95% CI: 0.881–0.978), and F1-score increased from 0.683 to 0.750. This pattern was consistent across 8 of 9 algorithms (Supplementary Table S1; Supplementary Figure S1). SHAP analysis of the 5-variable model identified Catheter-Related Complications (mean |SHAP| = 1.435) and Peritonitis (1.279) as the dominant predictors, followed by SSRS Score (0.699), PD Vintage (0.464), and Age (0.380) (Supplementary Figure S2). These findings demonstrate that the model’s discriminative power does not depend on SAS inclusion, and the clinical utility of the screening system extends beyond mere redundancy with anxiety screening. The 5-variable model, relying solely on clinical and psychosocial factors, may be more practical for point-of-care screening where concurrent anxiety assessment is unavailable. Detailed results are provided in Supplementary File 3.

### 3.10 Results of structural equation modeling (SEM) analysis

#### 3.10.1 Model specification

Based on the core research hypotheses and the six key predictors screened by LASSO regression, a structural equation model (SEM) was established to quantify the direct associations of each variable with elevated depressive symptoms (SDS) of peritoneal dialysis patients and the potential interrelationships among variables. The endogenous variable was elevated depressive symptoms (SDS), defined as a binary variable (0 = no elevated depressive symptoms, 1 = elevated depressive symptoms). The exogenous variables included age (0 = <60 years old, 1 = ≥60 years old), Peritonitis (0 = no, 1 = yes), Catheter-Related Complications (0 = no, 1 = yes), Self-Rating Anxiety Scale (SAS) score (0 = <50 points, 1 = ≥50 points), Social Support Rating Scale (SSRS) score (0 = <20 points, 1 = 20–29 points, 2 = ≥30 points), and Peritoneal Dialysis Vintage (PDV, a continuous variable in years). According to clinical logic and Spearman correlation analysis, covariance associations were specified between Peritonitis And Catheter-Related Complications as well as between SAS and SSRS. All exogenous variables directly predicted the endogenous variable SDS, constituting a saturated structural model. Since elevated depressive symptoms was a binary endogenous variable, the robust weighted least square mean and variance adjusted (WLSMV) estimator, which is specifically recommended for categorical and binary outcomes in SEM, was adopted to guarantee appropriate parameter estimation and valid statistical inference.

#### 3.10.2 Model fit assessment

The robust weighted least square mean and variance adjusted (WLSMV) estimator was used for model fitting, which is the standard and appropriate method for binary and non-normally distributed outcome variables in structural equation modeling. All fit indices met the ideal standards, indicating that the model was well-adapted to the data (Table 4).

**Table 4:** Fit indices of the structural equation model.

Fit indices	Value	Reference criteria
*χ*^2^ value	18.763	-
Degrees of freedom (df)	14	-
*χ*^2^/df	1.340	1 ~ 3 (good fit)
Root mean square error of approximation (RMSEA)	0.028	<0.08 (good fit)
Comparative Fit Index (CFI)	0.992	>0.90 (good fit)
Tucker-Lewis Index (TLI)	0.987	>0.90 (good fit)
Standardized root mean square residual (SRMR)	0.031	<0.08 (good fit)

Notably, the model is nearly saturated (df = 14), which typically results in excellent fit indices. Therefore, fit results should be interpreted with caution, and path coefficients should be evaluated primarily for clinical plausibility and consistency with SHAP/stratified analyses.

#### 3.10.3 Path coefficient analysis

The results of the direct associations of exogenous variables with elevated depressive symptoms (SDS) showed (Table 5) that 5 out of the 6 key factors were statistically significant (*p* < 0.05), and the direction of the associations was consistent with clinical cognition and SHAP analysis result.

**Table 5:** Standardized path coefficients and significance tests of the structural equation model.

Path (exogenous variable → SDS)	Unstandardized coefficient (est.)	Standard error (SE)	Standardized coefficient (std. est.)	*p*-value
Age (≥60)	0.327	0.114	0.095	0.004
Peritonitis (yes)	0.892	0.136	0.258	<0.001
Catheter-related complications (yes)	0.675	0.142	0.194	<0.001
Anxiety status (SAS ≥ 50)	0.783	0.129	0.226	<0.001
Social support (SSRS ≥ 30)	−0.541	0.153	−0.156	<0.001
PDV	0.021	0.018	0.037	0.041

#### 3.10.4 Covariance analysis of exogenous variables

All the set covariance associations between variables were statistically significant (Table 6), further verifying the internal correlation logic of clinical variables.

**Table 6:** Covariance estimation results of exogenous variables.

Associated variable pair	Covariance coefficient (est.)	Standard error (SE)	*p*-value
Peritonitis ↔ catheter-related complications	0.412	0.087	<0.001
SAS ↔ SSRS	−0.386	0.092	<0.001

#### 3.10.5 Model interpretation

The SEM constructed in this study clearly quantified the direct association intensity of each key factor with the risk of elevated depressive symptoms in peritoneal dialysis patients: peritonitis history had the most significant association (standardized coefficient = 0.258), followed by anxiety status (0.226) and catheter-related complications (0.194), all of which were risk factors for elevated depressive symptoms. In contrast, high-level social support (SSRS ≥30) showed a significant protective association (standardized coefficient = −0.156); age ≥60 years old had a weak positive association with risk (0.095), and peritoneal dialysis vintage (PDV) also exerted a weak positive association with risk (standardized coefficient = 0.037, *p* = 0.041), which was statistically significant.

This weak direct association of PDV is not contradictory to its predictive contribution in SHAP analysis and the significant association in stratified analysis in this study, as SHAP feature importance reflects model predictive contribution, stratified prevalence analysis is univariate correlation analysis, and SEM is multivariate association analysis with confounding factors adjusted.

In addition, the model verified the positive association between Peritonitis and Catheter-Related Complications (covariance coefficient = 0.412), suggesting that the two types of complications may interact or share common risk factors. The negative association between anxiety status and social support (covariance coefficient = −0.386) is consistent with psychosocial medical logic, i.e., insufficient social support may exacerbate anxiety. The model has excellent fit and results highly consistent with SHAP analysis, further confirming the direction and intensity of the associations of key predictors, and providing a more precise basis for clinical interventions.

## 4 Discussion

This investigation sought to determine primary factors associated with elevated depressive symptoms and to establish a dependable screening instrument for individuals receiving peritoneal dialysis (PD). Our principal outcomes indicate that age, occurrence of peritonitis, catheter-associated issues, scores on the Self-Rating Anxiety Scale (SAS), scores on the Social Support Rating Scale (SSRS), and duration of peritoneal dialysis (PDV) serve as independent factors associated with (not causally predictive of) elevated depressive symptoms, as this cross-sectional design cannot establish causality. These findings are consistent with recent epidemiological evidence that multi-dimensional risk factors are critical for depression screening in CKD patients with cardiometabolic comorbidities (5, 6), as PD patients’ elevated depressive symptoms are not only potentially driven by underlying cardiometabolic abnormalities, but also by dialysis-specific clinical complications and psychosocial stressors that are unique to this population. Of the nine machine learning models developed, the XGBoost model demonstrated superior efficacy, attaining an external validation AUC of 0.869 (95%CI: 0.775–0.947) and *F*-measure of 0.63. The external cohort’s clinical profile was broadly similar to the training cohort, supporting the validity of the external validation; however, the limited number of events (*n* = 24) warrants cautious interpretation. The resulting R Shiny-based online visualization tool showed favorable calibration and practical clinical utility within the studied populations. Further analyses through stratification and structural equation modeling (SEM) reinforced the consistency of these results within the studied cohorts, offering an evidence-based foundation for early detection and focused management of elevated depressive symptoms. Nevertheless, external validation in more heterogeneous clinical settings remains essential. These findings are consistent with the increasing acknowledgment that psychological comorbidities, especially depression and anxiety, are common in end-stage renal disease (ESKD) patients and substantially affect treatment compliance, life quality, and clinical outcomes (14).

Regarding the relationship between SAS and SDS: The SAS scale was adopted to assess anxiety symptoms, while the SDS scale was exclusively used to define the outcome of elevated depressive symptoms. Although anxiety and depression show high comorbidity in clinical practice, they represent distinct psychological constructs—anxiety is featured by persistent worry and hyperarousal, while depression is dominated by anhedonia and low mood (14), which constitutes the conceptual basis for treating SAS as an independent predictor and SDS as the outcome indicator in our model. Spearman correlation analysis in this study confirmed only a moderate correlation between SAS and SDS (*r* = 0.52–0.58) in PD patients, with no severe multicollinearity (|r| < 0.7), thus ruling out the risks of information overlap and same-source measurement bias. Clinically, anxiety is a well-established independent precursor of depression in ESKD/PD populations (14, 15). PD patients endure persistent physical stress from dialysis and its complications, and anxiety symptoms usually emerge earlier than depressive symptoms in this population; chronic anxiety can reduce patients’ stress tolerance and exacerbate their negative perception of the disease, thereby increasing the risk of subsequent depression. This clinical characteristic validates the rationality of including SAS as a core predictive factor, which enables the screening model to capture early psychological risk signals that cannot be reflected by SDS and further enhances its clinical value for early screening. In terms of model interpretation, the inclusion of SAS did not artificially inflate model performance but effectively enriched the interpretability of the screening system. SHAP analysis ranked SAS as the sixth core predictor affecting risk, and SEM results further quantified its significant direct positive association with elevated depressive symptoms (standardized coefficient = 0.226, *p* < 0.001), clarifying the intensity and direction of anxiety’s associative role in PD patients. Moreover, SEM covariance analysis confirmed a significant negative correlation between SAS and SSRS (covariance coefficient = −0.386, *p* < 0.001), which revealed the interactive mechanism between psychological factors (anxiety) and social factors (social support) in the pathogenesis of elevated depressive symptoms in PD patients. This finding extends the model’s interpretability from single-factor prediction to the interplay of multiple factors, and provides a clear clinical basis for targeted intervention—early management of anxiety symptoms in PD patients with high SAS scores can effectively reduce their subsequent risk of elevated depressive symptoms.

The recognition of Peritonitis and Catheter-Related complications as the foremost predictive elements is supported by clinical experience and recent investigations, which have associated PD-related complications with increased psychological strain and inflammatory pathways that influence mood (16, 17). Physical discomfort from peritonitis, recurrent medical procedures, and apprehension regarding treatment failure not only directly intensify psychological load but also interplay with catheter-related problems—these latter concerns interfere with daily activities and weaken confidence in treatment, together raising the likelihood of elevated depressive symptoms in PD patients (18, 19). Age of 60 years or older was identified as another crucial risk factor, mirroring the reduced physiological capacity, cognitive susceptibility, and lower stress tolerance in older adults patients on long-term PD. This corresponds with research documenting a greater incidence of depression among older ESKD populations (15). The strong positive association between an SAS score of 50 or higher and risk underscores the close comorbidity of anxiety and elevated depressive symptoms in these patients, as anxiety often precedes and intensifies depressive manifestations. This finding is supported by growing evidence that anxiety disorders impact a considerable segment of ESKD patients and involve shared neurobiological pathways with depression, including the chronic inflammation pathways also implicated in cardiometabolic disorder progression (5, 6).

Significantly, an SSRS score of 30 or above provided a notable protective influence, confirming that adequate social support alleviates stress related to dialysis and promotes psychological resilience. This aligns with contemporary studies highlighting social support as a key mediator connecting adverse clinical results to mental health in ESKD patients (20). Although PDV of 5 years or more showed a relatively modest effect size in SEM (*p* = 0.041), stratified prevalence analysis further verified its cumulative impact—likely due to extended physical discomfort, financial pressure, and treatment-associated exhaustion—which agrees with recent studies identifying longer dialysis duration as a consistent risk factor for depression (21). Beyond the clinical relevance of the identified predictors, the methodological approach of this study presents clear benefits. This study references the methodological framework of recent machine learning-based depression prediction models in cardiometabolic CKD populations, which have verified the value of integrating clinical and psychological factors for risk prediction (7), and further optimized this framework by incorporating PD-specific clinical indicators (e.g., Peritonitis, Catheter-Related Complications) and social support factors, making the model more tailored to the PD population with high cardiometabolic-renal comorbidity. In contrast to conventional logistic regression models, advanced machine learning algorithms like XGBoost more effectively capture non-linear relationships and potential interactions among variables while minimizing overfitting. The incorporation of LASSO variable selection, 5-fold cross-validation, and external validation (using a 7:3 cohort split) improves the model’s generalizability and dependability. The combination of Shapley Additive Explanations (SHAP) analysis and SEM tackles the “black box” limitation of machine learning by quantifying feature contributions and uncovering plausible associative pathways (e.g., the direct association of anxiety with elevated depressive symptoms. Notably, our sensitivity analysis (Section 3.9) demonstrated that the model’s discriminative performance remained robust—even improved—after excluding SAS, confirming that the screening utility extends beyond shared anxiety-depression variance and the protective function of social support), a methodological synthesis that is increasingly appreciated but still insufficiently documented in PD depression research (22, 23). Unlike single-center studies with restricted external validity, the multi-center design of this work enhances the online visualization tool’s applicability across varied clinical environments, addressing a significant gap in existing literature.

This investigation also introduces several noteworthy innovations. First, the systematic integration of clinical (peritonitis, catheter issues, PDV), psychological (anxiety), and social (support) factors offers a holistic biopsychosocial evaluation of risk, whereas earlier studies frequently concentrated on a narrower range of variables and rarely combined these dimensions with cardiometabolic considerations (5, 7). Second, the developed online visualization tool transforms complex model outputs into an accessible, points-based tool that demands no specialized statistical expertise, enabling routine clinical screening. This is especially valuable given the ongoing underdiagnosis of depression in dialysis contexts (24). Third, the congruence among SHAP analysis (for feature importance), stratified analysis (for subgroup effects), and SEM (for pathways) bolsters the reliability of the findings, ensuring that the identified predictors are not merely statistical artifacts but hold clinical and theoretical significance. These innovations respond to the pressing need for practical, evidence-based instruments to manage mental health in PD patients, as underscored in recent clinical guidelines advocating routine psychological screening for ESKD patients (14).

Despite these strengths, several limitations should be noted. First, some key clinical variables associated with elevated depressive symptoms were not included, such as dialysis adequacy (Kt/V), inflammatory markers (CRP and IL-6), nutritional status, psychiatric history, antidepressant use, and core cardiometabolic indicators (e.g., blood pressure, blood glucose, lipid profiles). Some of these variables (e.g., echocardiographic parameters, prealbumin) were excluded due to high missingness rates, while others showed no significant univariate association with SDS scores. The absence of cardiometabolic parameters, in particular, may limit the comprehensiveness and further optimization of the model, as these factors are well-documented to modulate depression risk in CKD populations (5, 6).

A post-hoc sensitivity analysis was not performed for these excluded variables with high missingness (Kt/V, prealbumin, echocardiographic parameters). Including these variables would have reduced the effective sample size by approximately 30–40% (from 601 to approximately 380–420), critically undermining the events per variable (EPV) requirement and model stability. Furthermore, multiple imputation at such high missingness rates (up to 32.7%) in a binary outcome with a relatively small sample may introduce substantial imputation bias and yield unreliable estimates. Given that preliminary univariate analysis showed no significant association between these variables and SDS scores, the potential incremental predictive value was considered insufficient to justify the methodological risks. Future studies with larger cohorts and prospectively collected complete data should evaluate whether incorporating these markers improves model performance.

Second, the retrospective design may lead to selection bias, and some unmeasured confounders could not be fully adjusted. Notably, while social support (SSRS) was included in the model, economic status (income level) was not captured. Financial strain is a well-documented driver of depression in chronic disease populations, and its absence may have led to residual confounding. Additionally, genetic predisposition and lifestyle factors were also unavailable (25).

Third, the relatively small number of events in the training cohort may restrict the complexity of machine learning models, although strict regularization was applied to reduce overfitting.

Fourth, the external validation cohort included only 119 patients with 24 positive events (SDS ≥ 53). Although the external cohort’s clinical profile was broadly similar to the training cohort, the limited number of events constrains the statistical power of external validation and results in wide confidence intervals for performance metrics. Further validation in larger, more diverse cohorts is warranted before broad clinical deployment.

Finally, this study used a concurrent cross-sectional design, which cannot establish temporal or causal relationships between predictors and outcomes. The identified factors should be interpreted as statistical associations rather than causal predictors; future prospective lagged studies are needed to validate the predictive value of these factors for incident depression and to determine whether modifying these factors would reduce depression risk.---.

Future prospective, multicenter studies with larger sample sizes are warranted to integrate the aforementioned clinical variables—including detailed cardiometabolic indicators and inflammatory markers (5, 6)—collect detailed psychiatric and treatment information, and further validate and optimize the Shiny tool (26). Expanding study populations and including more potential predictors such as nutritional markers, genetic polymorphisms, and digital biomarkers may also improve model performance (25). Randomized controlled trials are needed to verify targeted interventions, especially integrated strategies covering peritonitis prevention, anxiety management, social support enhancement, and cardiometabolic risk control, which may jointly reduce risk of elevated depressive symptoms in PD patients. Clinically, the Shiny tool could be integrated into routine PD follow-up to identify high-risk patients and deliver personalized care. Interdisciplinary cooperation between nephrologists, nurses, mental health professionals, and cardiometabolic specialists is also encouraged to improve comprehensive care for PD patients, addressing both physical and psychological comorbidities.

In summary, this study identifies six key predictors of elevated depressive symptoms in PD patients and develops an XGBoost-based online visualization tool with promising clinical applicability, though further validation in more diverse populations is warranted. The findings confirm the multifactorial nature of this condition in this population and emphasize the importance of integrating clinical, psychological, and social factors in risk assessment, while also highlighting the potential need to incorporate cardiometabolic indicators in future model optimization (5, 7). By providing a practical screening tool and clear intervention targets, this research contributes to the implementation of “biopsychosocial” integrated care for PD patients, which is essential for reducing incidence of elevated depressive symptoms, improving quality of life, and alleviating the overall healthcare burden of ESKD management.

## Data Availability

The original contributions presented in the study are included in the article/Supplementary material, further inquiries can be directed to the corresponding author.

## Glossary

AUC: Area under the receiver operating characteristic curve
APD: Automated peritoneal dialysis
CAPD: Continuous ambulatory peritoneal dialysis
CKD: Chronic kidney disease
DUV: Dialysate ultrafiltration volume
ESKD: End-stage renal disease
IL-6: Interleukin-6
ISPD: International Society for Peritoneal Dialysis
KDOQI: Kidney Disease Outcomes Quality Initiative
Kt/V: Dialysis adequacy measure
LASSO: Least absolute shrinkage and selection operator
ML: Machine learning
PD: Peritoneal dialysis
PDOPPS: Peritoneal Dialysis Outcomes and Practice Patterns Study
PDM: Peritoneal dialysis modality
PDV: Peritoneal dialysis vintage
PET: Peritoneal equilibrium test
SAS: Self-Rating Anxiety Scale
SDS: Self-Rating Depression Scale
SEM: Structural equation modeling
SHAP: Shapley additive explanations
SSRS: Social Support Rating Scale
WLSMV: Weighted least square mean and variance adjusted
XGBoost: Extreme gradient boosting
EPV: Events per variable.
