# Supplementary material for: Comparison and validation of machine learning-based screening models for elevated depressive symptoms in peritoneal dialysis patients
Source: Front Public Health. 2026 Jun 24;14:1792557. doi: 10.3389/fpubh.2026.1792557 (PMC13341524; doi:10.3389/fpubh.2026.1792557)
Supplement: Supplementary file 1 [file Supplementary_file_1.docx]

**Follow-up Standards for Peritoneal Dialysis Patients**

| Module | Specific Items | Detailed Content/Standards | Frequency/Applicable Situations |  |  |
| --- | --- | --- | --- | --- | --- |
| I. Core Principles of Follow-up | Stratified Follow-up | Routine follow-up for stable patients and intensive follow-up for high-risk/unstable patients to avoid excessive follow-up or missed risks | Applicable throughout the whole process |  |  |
|  | Whole-process Management | Covering the whole cycle of pre-dialysis preparation, post-catheterization, dialysis initiation, maintenance period, and outcome (transfer to hemodialysis/renal transplantation/death) | Applicable throughout the whole process |  |  |
|  | Multidisciplinary Collaboration | Led by nephrology department, combined with nutrition department, surgery department (for hernia/catheter-related complications), psychology department, etc. | Applicable throughout the whole process, with priority initiation during the complication period |  |  |
| II. Follow-up Frequency | (I) Pre-dialysis Period (from post-catheterization to dialysis initiation) | First follow-up after catheterization | Bedside follow-up 24-48 hours after surgery |  |  |
|  |  | Follow-up for wound dressing change and suture removal | Outpatient follow-up 7-10 days after surgery |  |  |
|  |  | Pre-dialysis initiation preparation follow-up | Outpatient/home follow-up 1-3 days before initiation |  |  |
|  | (II) Dialysis Initiation Period (1-3 months) | Follow-up during novice adaptation period | Twice a week outpatient follow-up or daily home remote follow-up within 1 week after initiation |  |  |
|  |  | Follow-up during prescription stabilization period | Weekly outpatient follow-up 2-4 weeks after initiation |  |  |
|  |  | Follow-up for transition to maintenance period | Outpatient follow-up every 2 weeks 2-3 months after initiation |  |  |
|  | (III) Dialysis Maintenance Period (after 3 months) | Follow-up for routine stable patients | Monthly outpatient follow-up and comprehensive assessment every 3 months |  |  |
|  |  | Follow-up for high-risk patients (complicated with diabetes, cardiovascular diseases, etc.) | Outpatient follow-up every 2 weeks |  |  |
|  | (IV) Unstable Condition/Complication Period | Follow-up for acute complications (peritonitis, intra-abdominal hemorrhage, etc.) | Immediate follow-up, hospitalization if necessary; weekly follow-up after condition control, and return to routine frequency if no recurrence for 2-4 consecutive weeks |  |  |
|  |  | Follow-up for chronic complications (renal anemia, secondary hyperparathyroidism, etc.) | Every 2 weeks until indicators meet the standard and stabilize for 1 month |  |  |
| III. Core Content of Follow-up | (I) Medical History Collection (Mandatory Items) | Dialysis-related history | Daily ultrafiltration volume, urine output, color/turbidity of peritoneal dialysate, abdominal pain or distension during fluid exchange; recent dialysis prescription adjustment history | Every follow-up (outpatient + home) |  |
|  |  | Systemic symptoms | Fever, fatigue, loss of appetite, edema, chest tightness and shortness of breath, nausea and vomiting, focusing on hernia-related symptoms (abdominal pain, abdominal mass, distension) | Every follow-up (outpatient + home) |  |
|  |  | Comorbidity history | Blood glucose control in diabetic patients, blood pressure fluctuation in hypertensive patients, chest pain and palpitations in patients with cardiovascular diseases | Every follow-up (outpatient + home) |  |
|  |  | Medication history | Verify compliance with antihypertensive drugs, erythropoietin, iron supplements, vitamin D analogs, etc., and whether there are newly added/discontinued drugs | Every follow-up (outpatient + home) |  |
|  | (II) Physical Examination | Basic vital signs | Blood pressure (supine + standing position, to check orthostatic hypotension), heart rate, body temperature, body weight (weighed on an empty stomach after emptying the abdominal cavity, core indicator) | Mandatory for each outpatient follow-up; daily self-examination of body weight and blood pressure at home |  |
|  |  | Abdominal examination | Abdominal circumference (measured once a week to check intra-abdominal effusion), presence of redness, swelling and exudation at the wound/exit site, presence of tenderness and rebound tenderness, presence of mass (focus on checking PD-related hernias, such as incisional hernia, umbilical hernia, inguinal hernia) | Mandatory for each outpatient follow-up; self-examination of abdominal circumference and wound at home once a week |  |
|  |  | Systemic examination | Edema degree (eyelids, lower limbs), cardiopulmonary auscultation (to check heart failure, pulmonary infection), skin and mucous membranes (presence of pallor and ecchymosis, to assess anemia) | Mandatory for each outpatient follow-up; self-examination of edema at home |  |
|  | (III) Laboratory Examinations | High-frequency Mandatory Items (each outpatient follow-up) | Routine blood test | Assess anemia (hemoglobin, hematocrit) and infection (white blood cell count) | Each outpatient follow-up |
|  |  |  | Electrolytes + Renal function | Serum potassium, sodium, chloride, calcium, phosphorus, creatinine, urea nitrogen to judge electrolyte disturbance and renal function status | Each outpatient follow-up |
|  |  |  | Blood glucose (for diabetic patients) | Fasting + 2-hour postprandial, or glycated hemoglobin (once every 3 months) | Each outpatient follow-up; glycated hemoglobin once every 3 months |
|  |  | Medium-frequency Items (once every 3 months, comprehensive assessment) | Dialysis adequacy | Urea Clearance Index (Kt/V) ≥ 1.7/week, Creatinine Clearance Rate (Ccr); according to KDOQI guidelines, complete the first assessment within 1 month after dialysis initiation, then monitor every 4 months, and supplement 24-hour urine assessment every 2 months for patients with residual renal function > 100ml/d | Once every 3 months |
|  |  |  | Nutritional indicators | Serum albumin (< 35g/L requires intervention), prealbumin, total protein | Once every 3 months |
|  |  |  | Anemia-specific indicators | Serum ferritin, transferrin saturation to guide the use of iron supplements | Once every 3 months |
|  |  |  | Bone metabolism indicators | Intact Parathyroid Hormone (iPTH 150-300pg/mL), serum calcium, serum phosphorus | Once every 3 months |
|  |  | Low-frequency Screening Items (once every 6-12 months, chronic disease management) | Liver function, blood lipids, coagulation function (for long-term anticoagulated patients), infectious disease screening (hepatitis B, hepatitis C, etc.) | Once every 6-12 months |  |
|  |  | (IV) Imaging and Special Examinations | Abdominal ultrasound | Assess intra-abdominal effusion, position of peritoneal dialysate catheter, organ morphology; clarify the type and size when hernia is suspected; according to KDOQI guidelines, complete baseline peritoneal function-related ultrasound assessment 4-8 weeks after dialysis initiation, then once every 3-6 months; immediate examination when hernia is suspected | Once every 3-6 months; immediate examination when hernia is suspected |
|  | Echocardiography |  | Assess cardiac function and ventricular hypertrophy | Once every 6-12 months; once every 3 months for patients with cardiovascular diseases |  |
|  | Chest X-ray |  | Screen for pulmonary infection and heart failure | Once a year; temporary examination when relevant symptoms appear |  |
|  | Peritoneal dialysate examination |  | Routine, bacterial culture + drug sensitivity to screen for peritonitis | Immediate submission for examination when peritoneal dialysate is turbid or abdominal pain occurs |  |
|  | (V) Health Guidance and Compliance Assessment | Operation guidance, dietary guidance, medication guidance, complication prevention, psychological assessment. Psychological status assessment should be conducted with Self-Rating Depression Scale (SDS) and Self-Rating Anxiety Scale (SAS) every 3-6 months. Corresponding psychological intervention measures should be provided according to the assessment results, and referral to psychiatry department for further evaluation and treatment should be made if necessary. | Every follow-up (outpatient + home), throughout the whole process; Psychological status assessment with SDS and SAS every 3-6 months |  |  |
|  | IV. Follow-up Record and Management | Unified Form | Fill in basic patient information, follow-up date, examination results, prescription adjustments, and guidance content | Mandatory for each follow-up |  |
|  |  | Information Retention | File outpatient records, keep written records of home follow-up, and ensure data traceability | Mandatory for each follow-up |  |
|  |  | Early Warning Mechanism | Timely mark and intervene when indicators are abnormal, such as hemoglobin < 110g/L, iPTH > 500pg/mL | Applicable throughout the whole process, immediate assessment after results are available |  |
|  |  | Outcome Record | Complete the final follow-up record when transferring to hemodialysis, renal transplantation, or death, indicating the cause and time | Mandatory when the patient's outcome occurs |  |
| V. Supplementary Follow-up Standards for Special Populations | Elderly patients (≥ 65 years old) | Simplify the process, increase the frequency of home follow-up, check cognitive function and operational ability, and arrange family accompaniment if necessary | At least once every 2 weeks of home follow-up and once a month of outpatient follow-up |  |  |
|  | Diabetic PD patients | Increase blood glucose monitoring (at least 4 times a week), check glycated hemoglobin every 3 months, and give priority to controlling blood pressure and blood glucose | Outpatient follow-up every 2 weeks, daily self-examination of blood glucose at home |  |  |
|  | PD patients with hernia | Check the size of the hernia mass and the risk of incarceration, guide abdominal belt compression, and avoid sudden increase of abdominal pressure | Recheck ultrasound every 1-2 months and follow up once every 2 weeks |  |  |
| VI. Follow-up Termination Standards | Termination Scenarios | 1. Successful renal transplantation with restored normal renal function; 2. Transfer to hemodialysis and stable for more than 3 months; 3. Patient death | Terminate when any scenario is met and complete the final follow-up record |  |  |
