# Supplementary material for: Comparison and validation of machine learning-based screening models for elevated depressive symptoms in peritoneal dialysis patients
Source: Front Public Health. 2026 Jun 24;14:1792557. doi: 10.3389/fpubh.2026.1792557 (PMC13341524; doi:10.3389/fpubh.2026.1792557)
Supplement: Supplementary file 2 [file Supplementary_file_2.docx]

Supplementary Material

**TRIPOD+AI Checklist for the Reporting of Prediction Model Studies**

| **Section/topic** | **Item** | **Development/evaluation** | **Checklist item** | **Verification results** | **Page/Section** |
| --- | --- | --- | --- | --- | --- |
| **Title** | 1 | D;E | Identify the study as developing or evaluating the performance of a multivariable prediction model, the target population, and the outcome to be predicted | This study develops and externally validates a machine learning screening model for elevated depressive symptoms in peritoneal dialysis patients. The target population is adult patients aged 18-75 years with end-stage renal disease (ESRD) receiving maintenance peritoneal dialysis. The outcome is elevated depressive symptoms defined as SDS score ≥53. | Title + Abstract |
| **Abstract** | 2 | D;E | See TRIPOD+AI for Abstracts checklist | The abstract fully reports the study purpose, design, methods, key results and conclusions, complying with TRIPOD+AI abstract guidelines. | Abstract |
| **Introduction** |  |  |  |  |  |
| **Background** | 3a | D;E | Explain the healthcare context (including whether diagnostic or prognostic) and rationale for developing or evaluating the prediction model, including references to existing models | Healthcare context: The prevalence of depression in peritoneal dialysis patients ranges from 27.6% to 45.0%, and current screening methods are highly subjective with high missed diagnosis rates. Existing machine learning models are limited by single-center designs, lack of external validation, and poor interpretability. | Section 1.1 Introduction |
|  | 3b | D;E | Describe the target population and the intended purpose of the prediction model in the context of the care pathway, including its intended users (eg, healthcare professionals, patients, public) | Target population: Adult patients with ESRD receiving maintenance peritoneal dialysis. Intended purpose: Rapid point-of-care screening during routine follow-up visits. Intended users: Nephrologists, peritoneal dialysis specialist nurses, and primary care providers. | Abstract + Section 3.7 |
|  | 3c | D;E | Describe any known health inequalities between sociodemographic groups | Baseline analysis showed that rural patients and patients with resident medical insurance had lower social support scores and higher depression risk. Elderly patients had significantly higher depression risk than younger patients. | Section 3.1 + Section 3.8 |
| **Objectives** | 4 | D;E | Specify the study objectives, including whether the study describes the development or validation of a prediction model (or both) | 1. Develop and validate a machine learning screening model for elevated depressive symptoms in peritoneal dialysis patients; 2. Identify core predictive factors and elucidate their associative pathways; 3. Develop a clinically practical online visualization tool. | Section 1.3 Study Objectives |
| **Methods** |  |  |  |  |  |
| **Data** | 5a | D;E | Describe the sources of data separately for the development and evaluation datasets (eg, randomised trial, cohort, routine care or registry data), the rationale for using these data, and representativeness of the data | Development + internal validation dataset: 475 peritoneal dialysis patients from Huangshi Central Hospital (tertiary A hospital) between February 2010 and April 2022. External validation dataset: 126 patients from Honghu People's Hospital (tertiary B hospital) during the same period. Data source: Hospital Information System (HIS). Representativeness: Covers peritoneal dialysis populations from different levels of hospitals in central China. | Section 2.1 |
|  | 5b | D;E | Specify the dates of the collected participant data, including start and end of participant accrual; and, if applicable, end of follow-up | Participant accrual period: February 2010 to April 2022. End of follow-up: April 2022. | Section 2.1 |
| **Participants** | 6a | D;E | Specify key elements of the study setting (eg, primary care, secondary care, general population) including the number and location of centres | Study setting: Nephrology departments and peritoneal dialysis centers of 2 tertiary hospitals. Location: Huangshi City and Honghu City, Hubei Province, China. | Section 2.1 |
|  | 6b | D;E | Describe the eligibility criteria for study participants | Inclusion criteria: Aged 18-75 years, confirmed diagnosis of ESRD, no history of malignant tumors, normal cognitive function (MMSE ≥24 or MoCA ≥26) able to complete scale assessments. Exclusion criteria: Incomplete clinical data, expected survival <6 months, participation in other interventional clinical trials within 3 months, pregnancy or lactation. | Sections 2.1.1-2.1.2 |
|  | 6c | D;E | Give details of any treatments received, and how they were handled during model development or evaluation, if relevant | All patients received standard peritoneal dialysis treatment (predominantly CAPD). Treatment regimens were not included as model variables. The model only used objectively measured clinical indicators and self-reported scale results obtained during follow-up. | Section 2.3 |
| **Data preparation** | 7 | D;E | Describe any data pre-processing and quality checking, including whether this was similar across relevant sociodemographic groups | Data pre-processing: All patient identifiers were anonymized. Quality checking: Double cross-validation of data entry accuracy. Missing data handling: Complete case analysis (missing rate <5% for all candidate variables). Processing procedures were identical across all age, gender, and urban-rural groups. | Beginning of Section 2.4 |
| **Outcome** | 8a | D;E | Clearly define the outcome that is being predicted and the time horizon, including how and when assessed, the rationale for choosing this outcome, and whether the method of outcome assessment is consistent across sociodemographic groups | Outcome: Elevated depressive symptoms (SDS ≥53). Assessment time: Measured concurrently with all predictors during routine follow-up visits (every 3-6 months). Rationale: SDS is an internationally validated depression screening scale with good reliability and validity. Scale administration procedures were fully standardized across both centers. | Section 2.2 + Study Design Clarification in Section 2.3 |
|  | 8b | D;E | If outcome assessment requires subjective interpretation, describe the qualifications and demographic characteristics of the outcome assessors | The outcome was assessed via patient self-report scales, administered and guided by uniformly trained peritoneal dialysis specialist nurses. All nurses received standardized training in scale administration. | Section 2.3 |
|  | 8c | D;E | Report any actions to blind assessment of the outcome to be predicted | All data were anonymized. Statistical analysts were blinded to patient clinical grouping and outcome status. Scale completion was independent of clinical treatment assessments. | Ethics Section in Section 2.1 |
| **Predictors** | 9a | D | Describe the choice of initial predictors (eg, literature, previous models, all available predictors) and any pre-selection process for predictors before model building | A total of 23 candidate predictors were initially selected based on literature review and clinical experience, covering 4 dimensions: demographic, clinical, dialysis-related, and psychosocial factors. Pre-selection: Variables with missing rate >5% were excluded, followed by core predictor selection via LASSO regression. | Section 2.2 + Section 3.4 |
|  | 9b | D;E | Clearly define all predictors, including how and when they were measured (and any blinding of predictor assessment to avoid bias, such as blinding to outcome and other predictors) | Core predictors: Age (at enrollment), history of peritonitis (number of episodes during follow-up), history of catheter-related complications (number of episodes during follow-up), SAS score (self-reported at follow-up), SSRS score (self-reported at follow-up), and peritoneal dialysis vintage (at enrollment). All predictor assessments were blinded to outcome status. | Section 2.2 + Section 2.3 |
|  | 9c | D;E | If predictor measurement requires subjective interpretation, describe the qualifications and demographic characteristics of the predictor assessors | Clinical indicators were obtained from objective laboratory tests. Scales were administered by trained specialist nurses with no subjective interpretation involved. | Section 2.3 |
| **Sample size** | 10 | D;E | Explain how the study sample size was determined (separately for development and evaluation phases) and justify that the sample size is sufficient to answer the research question. Include details of any sample size calculations performed | Sample size justification: (1) 10 events per variable (EPV) rule: 6 predictors require 60 events; our cohort included 116 total events. (2) Riley et al. (2020) guidelines: ≥20 events are sufficient for preliminary external validation; our external validation cohort included 20 events. | Section 2.1.3 |
| **Missing data** | 11 | D;E | Describe the method used to handle missing data and justify any exclusion of data | **Complete case analysis** was used. Justification: Missing rates for all candidate variables ranged from 0.2% to 3.8%, below the 5% threshold for significant bias. No imputation was performed to preserve the authenticity of clinical data. | Beginning of Section 2.4 |
| **Analytical methods** | 12a | D | Describe how the data were used in the analysis (eg, for model development and performance evaluation), including whether data were grouped and whether sample size requirements were considered | Data from Huangshi Central Hospital were randomly split into training set (n=356) and internal validation set (n=119) at a 7:3 ratio. Data from Honghu People's Hospital served as an independent external validation set (n=126). All groupings met the EPV ≥10 requirement. | Section 2.1.3 |
|  | 12b | D | Describe how predictors were handled in the analysis (eg, functional form, rescaling, transformation, or any standardisation methods) according to the model type | Continuous variables were retained in their original form. Binary variables were coded as 0/1. Categorical variables were dummy-coded. Tree-based models (XGBoost, RF, etc.) did not require standardization. | Section 2.4 |
|  | 12c | D | Specify the model type, rationale for choosing this model, all model building steps (including any hyperparameter tuning), and methods for internal validation | Nine machine learning models were constructed (LR, ENet, DT, RF, XGBoost, LightGBM, MLP, RSVM, KNN). Rationale: Comprehensive comparison of performance across different algorithms. Modeling steps: LASSO variable selection → stratified 5-fold cross-validation → grid search hyperparameter tuning. Internal validation: 5-fold cross-validation + independent internal validation set. | Hyperparameter Section in Section 2.4 |
|  | 12d | D;E | Describe whether and how heterogeneity in model parameter values and estimates of model performance between different clusters (eg, hospitals, countries) was addressed and quantified. See TRIPOD-Cluster for additional considerations‡ | Heterogeneity across centers was evaluated via independent external validation. Despite significant baseline differences between the external validation set and training set, the XGBoost model still demonstrated excellent performance, indicating good cross-center generalizability. | External Validation Discussion in Section 3.5 |
|  | 12e | D;E | Specify all metrics and plots used to evaluate model performance (eg, discrimination, calibration, clinical utility) and their rationale; if applicable, also describe metrics and plots used to compare multiple models | Performance metrics: AUC, accuracy, sensitivity, specificity, precision, recall, F1-score. Calibration metrics: Calibration curves, Brier score, calibration slope, calibration intercept. Clinical utility: Decision Curve Analysis (DCA). Rationale: Comprehensive assessment of model discrimination, calibration, and clinical practical value. | Section 2.4 + Section 3.5 |
|  | 12f | E | Describe whether model updating (eg, recalibration) was performed after model evaluation, including overall updating or updating for specific sociodemographic groups or settings | No model updating was performed. This is the first development and external validation of the model. Future updates will be based on larger samples. | - |
|  | 12g | E | For model evaluation, describe how model predictions were calculated (eg, formula, code, object, application programming interface) | Model predictions are implemented via the publicly available R Shiny online tool (<https://caoyugang.shinyapps.io/DepressionRiskPrediction/>), which requires no programming skills. Complete analysis code is available upon request from the corresponding author. | Section 3.7 |
| **Class imbalance** | 13 | D;E | If a class imbalance approach was used, explain the rationale and specific operations of this method, as well as any subsequent methods used to recalibrate the model or model predictions | **Stratified 5-fold cross-validation** was used to address class imbalance (event rate ≈20%). Rationale: Ensures the proportion of positive events in each fold matches the overall dataset. No class weighting or resampling was applied, as stratified cross-validation sufficiently stabilized performance estimates. | Section 2.4 |
| **Fairness** | 14 | D;E | Describe any methods used to address model fairness and the rationale for their selection | Model performance across subgroups (age, gender, peritonitis history, dialysis vintage, social support, anxiety status) was evaluated via **stratified cumulative risk analysis**. Risk differences were statistically significant across all subgroups (all P<0.05), and the model demonstrated good discrimination in all subgroups. | Section 3.8 |
| **Model output** | 15 | D | Specify the output of the prediction model (eg, probability, classification result). Provide details of any classification, the method used to determine the classification threshold, and its rationale | Model output: Probability of elevated depressive symptoms ranging from 0 to 1. Default classification threshold: 0.5 (balances sensitivity and specificity). Risk stratification thresholds: <20% (low risk), 20%-50% (moderate risk), 50%-80% (moderate-high risk), >80% (high risk). Stratification rationale: Prioritizes high sensitivity to minimize missed diagnoses. | Section 2.4 + Section 3.7 |
| **Comparison of training and evaluation** | 16 | D;E | Specify any differences between the development and evaluation datasets in terms of healthcare setting, eligibility criteria, outcome measures, and predictors | Baseline differences between external validation set and training set: Younger (70.6% <60 years vs 37.1%), more females (56.3% vs 41.0%), lower diabetes prevalence (15.1% vs 27.8%), lower albumin levels (33.6 vs 37.4 g/L), higher social support scores (42.9% ≥30 vs 14.0%), fewer catheter complications (28.6% vs 45.5%). | Section 3.1 |
| **Ethical approval** | 17 | D;E | State the name of the institutional review board or ethics committee that approved the study, and describe the informed consent status of participants or the ethics committee's waiver of informed consent | Approving bodies: Institutional Review Board of Huangshi Central Hospital (Lun Kuai Shen [2025]-48) and Medical Ethics Committee of Honghu People's Hospital (HHRY[2025]-45). Informed consent: Written informed consent was waived for this retrospective study. | Section 6 Ethics Statement |
| **Open science** |  |  |  |  |  |
| **Funding support** | 18a | D;E | State the source of funding for the study and the role of the funder(s) in the study | Funding source: Open Fund Project of Hubei Provincial Key Laboratory of Kidney Disease Pathogenesis and Intervention (Project No.: 2024SJ107). Funder role: No involvement in study design, data collection, analysis, or manuscript writing. | Section 8 Funding |
| **Conflicts of interest** | 18b | D;E | Disclose any conflicts of interest and financial disclosures for all authors | All authors declare no relevant conflicts of interest. | Section 9 Conflict of Interest |
| **Study protocol** | 18c | D;E | State where the study protocol is available, or declare that no study protocol was developed | No formal study protocol was pre-specified. | - |
| **Study registration** | 18d | D;E | Provide study registration details, including the registration platform name and registration number, or declare that the study was not registered | This study was not registered on a clinical trial platform. | - |
| **Data sharing** | 18e | D;E | Provide detailed information on the availability of study data | Original data are included in this article and supplementary materials. Further data inquiries can be directed to the corresponding author. | Section 5 Data Availability Statement |
| **Code sharing** | 18f | D;E | Provide detailed information on the availability of analytical code§ | Complete data analysis code is available upon request from the corresponding author via email. The online tool code is deployed on the Shiny server and publicly accessible. | Section 3.7 |
| **Patient and public involvement** | 19 | D;E | Describe the involvement of patients and the public in the study design, conduct, reporting, interpretation, or dissemination, or declare that there was no such involvement | No patient or public involvement in this study. | - |
| **Results** |  |  |  |  |  |
| **Participants** | 20a | D;E | Describe the flow of participants through the study, including the number of participants with and without outcome events; if applicable, also summarise follow-up time. A flowchart is recommended to assist in illustration | Total eligible patients: 601. Training set: 356 patients (72 events, 20.2%); internal validation set: 119 patients (24 events, 20.2%); external validation set: 126 patients (20 events, 15.9%). Median follow-up time: 4.0 years. A patient enrollment flowchart is recommended as Supplementary File 3. | Section 2.1.3 + Section 3.1 |
|  | 20b | D;E | Report the overall characteristics of participants, and if applicable, separately for each data source or setting, including key dates, key predictors (including demographic characteristics), treatments received, sample size, number of outcome events, follow-up time, and amount of missing data. A table is recommended to assist in illustration. Differences between key demographic groups should be reported | Baseline characteristics of all cohorts are detailed in Table 1, including age, gender, marital status, education level, insurance type, residence, dialysis-related indicators, and psychological scale scores. Baseline differences across key demographic groups are clearly reported. | Section 3.1 + Table 1 |
|  | 20c | E | For model evaluation, show the distribution comparison of key predictors (demographic characteristics, predictors, and outcome measures) between the development and evaluation datasets | Comparison of baseline characteristics between the development set and external validation set is detailed in Table 1. Distribution differences for all key variables are reported. | Section 3.1 + Table 1 |
| **Model development** | 21 | D;E | Specify the number of participants and outcome events in each analysis (eg, model development, hyperparameter tuning, model evaluation) | Training set: 356 patients / 72 events; internal validation set: 119 patients / 24 events; external validation set: 126 patients / 20 events. Hyperparameter tuning was performed within the 5-fold cross-validation of the training set. | Section 2.1.3 + Section 3.5 |
| **Model specification** | 22 | D | Provide detailed information on the complete prediction model (eg, formula, code, object, application programming interface) to enable prediction in new individuals and support third-party evaluation and implementation, including any access or reuse restrictions (eg, freely available, proprietary copyright)¶ | The complete model is publicly available via the R Shiny online tool (<https://caoyugang.shinyapps.io/DepressionRiskPrediction/>). No access restrictions, free for non-commercial use. Complete code is available upon request from the corresponding author. | Section 3.7 |
| **Model performance** | 23a | D;E | Report estimates of model performance with confidence intervals, including results for any key subgroups (eg, sociodemographic subgroups). A plot is recommended to assist in presentation | XGBoost model performance in external validation (95% CI): Accuracy 0.93 (0.88-0.97), sensitivity 0.91 (0.78-0.98), specificity 0.87 (0.80-0.93), F1-score 0.86 (0.76-0.93). Subgroup performance is detailed in Section 3.8 Stratified Analysis. | Section 3.5 + Section 3.8 |
|  | 23b | D;E | If relevant analyses were performed, report the results of heterogeneity in model performance between different clusters. See TRIPOD-Cluster for additional details‡ | The F1-score in the external validation set (0.86) was superior to that in the internal validation set (0.73), potentially due to lower event rate, simpler case mix, and more standardized scale administration. No significant performance heterogeneity was observed. | Section 3.5 |
| **Model updating** | 24 | E | Report the results of any model updating, including the updated model and its subsequent performance | No model updating was performed. | - |
| **Discussion** |  |  |  |  |  |
| **Interpretation of results** | 25 | D;E | Provide an overall interpretation of the main results, including a discussion of fairness issues in the context of the study objectives and previous research | Key findings: The XGBoost model achieved optimal performance, the 6 core predictors have clear clinical significance, and the online tool has good clinical utility. Results are consistent with existing literature. The model demonstrated stable performance across all subgroups with no significant fairness issues. | Section 4 Discussion |
| **Study limitations** | 26 | D;E | Discuss any limitations of the study (eg, unrepresentative sample, insufficient sample size, overfitting, missing data) and their impact on bias, statistical uncertainty, and generalisability | Limitations: Retrospective design, some potential variables excluded due to high missing rates, relatively small external validation sample size, concurrent design cannot establish causality, lack of prospective validation. | Section 4.4 Limitations |
| **Applicability of the model in current healthcare** | 27a | D | Describe how poor-quality or unavailable input data (eg, predictor values) will be assessed and handled when implementing the prediction model | The online tool requires complete input of all 6 core variables. Missing values must be supplemented by clinical staff before calculation. No automatic imputation is supported to ensure prediction accuracy. | Section 3.7 |
|  | 27b | D | Specify whether user interaction is required when processing input data or using the model, and the level of professional knowledge required of the user | User interaction: Simple form input, click "Calculate" to obtain results. Professional knowledge requirement: No statistical or programming skills needed. Clinical staff can use it after brief training. | Section 3.7 |
|  | 27c | D;E | Discuss future research plans, focusing on the applicability and generalisability of the model | Future plans: 1. Conduct prospective multicenter large-sample validation; 2. Incorporate more clinical and biological markers; 3. Evaluate the clinical intervention effect of the tool; 4. Optimize the model for specific populations. | Section 4.5 Future Research Directions |

**Notes:**

- D: Applicable only to prediction model development studies;
- E: Applicable only to prediction model evaluation studies;
- D;E: Applicable to both prediction model development and evaluation studies.
- The rationale for choosing all model building methods should be explained separately.
- TRIPOD-Cluster is a reporting checklist developed for prediction model development or validation studies that explicitly consider clustering effects or explore heterogeneity in model performance (eg, different hospitals or centres).
- Refers to analytical code, such as any code related to data cleaning, feature engineering, model building, and evaluation.
- Refers to model implementation code used to obtain risk estimates in new individuals.
