# Supplementary figures and images for: Comparison and validation of machine learning-based screening models for elevated depressive symptoms in peritoneal dialysis patients
Source: Front Public Health. 2026 Jun 24;14:1792557. doi: 10.3389/fpubh.2026.1792557 (PMC13341524; doi:10.3389/fpubh.2026.1792557)

Supplementary Material

**Patient Enrollment Flowchart**

**
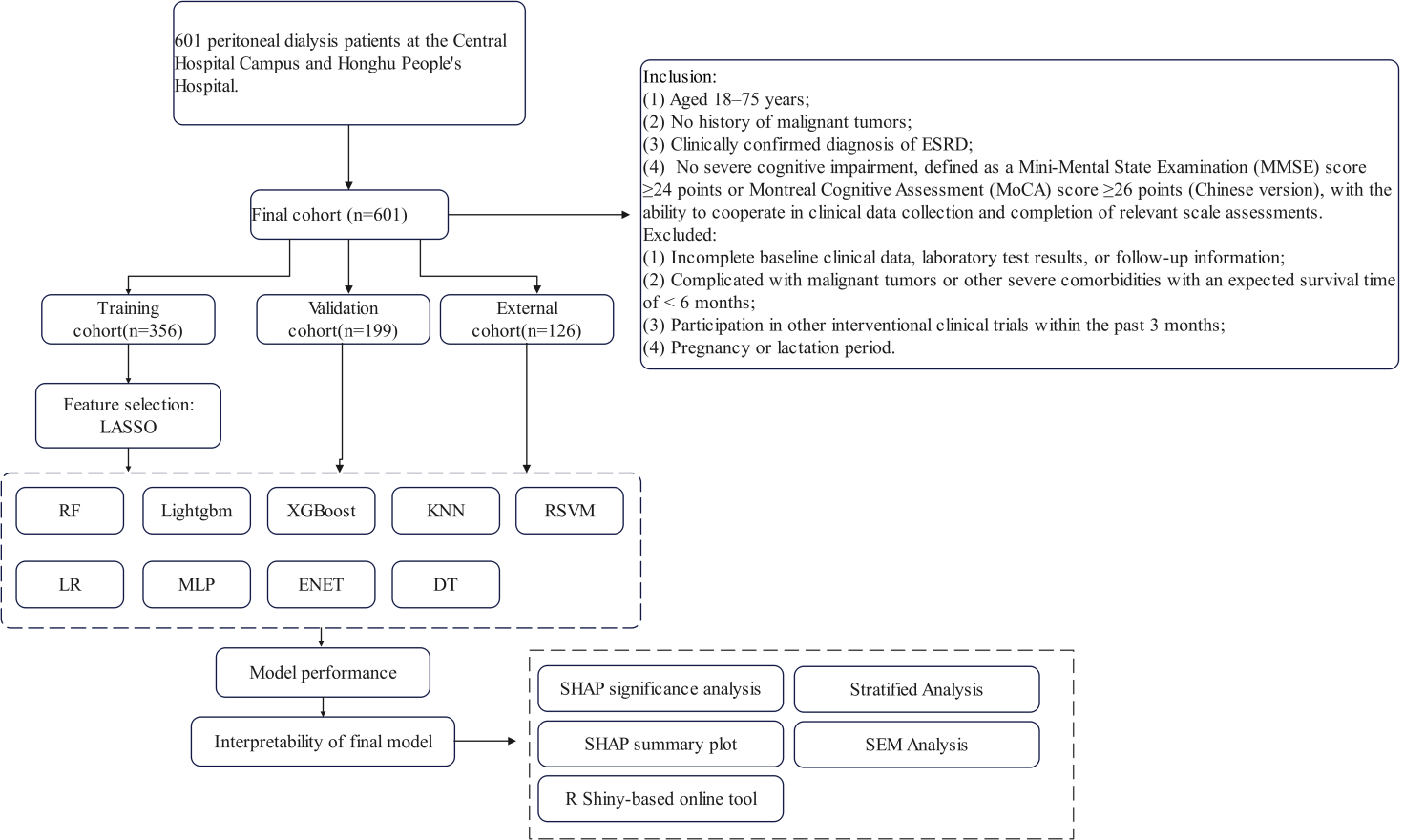
**

Supplement: Supplementary file 3 [file Supplementary_file_3.docx]
