# Supplementary material for: Comparison and validation of machine learning-based screening models for elevated depressive symptoms in peritoneal dialysis patients
Source: Front Public Health. 2026 Jun 24;14:1792557. doi: 10.3389/fpubh.2026.1792557 (PMC13341524; doi:10.3389/fpubh.2026.1792557)
Supplement: Supplementary file 4 [file Supplementary_file_4.docx]

Supplementary Material

**Supplementary File: Sensitivity Analysis — Excluding Self-Rating Anxiety Scale (SAS)**

**1. Rationale**

The reviewer raised a legitimate concern regarding potential information leakage arising from the inclusion of the Self-Rating Anxiety Scale (SAS) as a predictor. Anxiety and depressive symptoms exhibit high comorbidity and significant item-level overlap (e.g., sleep disturbances, concentration difficulties, fatigue). In our sample, the Spearman correlation between SAS and SDS scores ranged from 0.52 to 0.58 across cohorts, and 75.0% of patients with elevated depressive symptoms (SDS ≥53) also had elevated anxiety scores (SAS ≥50). Including SAS as a predictor may therefore inflate model performance through shared source variance rather than genuine discriminative power, wherein the model essentially predicts "psychological distress" using concurrent "psychological distress." This sensitivity analysis was conducted to determine whether the model's utility extends beyond mere redundancy with existing anxiety screening tools.

**2. Methods**

An alternative 5-variable XGBoost model was constructed by excluding SAS, retaining: Age (<60 vs. ≥60 years), Peritonitis (no vs. yes), Catheter-Related Complications (no vs. yes), SSRS Score (<20 vs. 20–29 vs. ≥30), and Peritoneal Dialysis Vintage (continuous, years). All nine machine learning algorithms were similarly retrained on the 5-variable set using identical hyperparameter tuning (5-fold stratified cross-validation with grid search) and evaluation procedures. Performance was compared with the original 6-variable models across internal validation (n=119) and external validation (n=119) cohorts. Bootstrap 95% confidence intervals (n=1000 resamples) were calculated for AUC. SHAP analysis was performed on the 5-variable XGBoost model to assess feature importance.

**3. Results**

**3.1 XGBoost Performance Comparison**

Contrary to the expectation that SAS removal would degrade performance, the 5-variable XGBoost model achieved comparable or superior performance on external validation:

**Table S1.** Performance comparison of 6-variable (with SAS) vs. 5-variable (excluding SAS) XGBoost models. AUC values are presented with bootstrap 95% CI (n=1000). IntVal = internal validation; ExtVal = external validation.

| Metric | 6-var IntVal | 5-var IntVal | 6-var ExtVal | 5-var ExtVal |
| --- | --- | --- | --- | --- |
| AUC | 0.881 (0.774–0.967) | 0.931 (0.879–0.973) | 0.869 (0.775–0.947) | 0.890 (0.810–0.956) |
| Accuracy | 0.874 | 0.866 | 0.897 | 0.921 |
| Sensitivity | 0.600 | 0.600 | 0.542 | 0.542 |
| Specificity | 0.953 | 0.953 | 0.958 | 0.947 |
| Precision | 0.706 | 0.706 | 0.765 | 0.722 |
| F1-score | 0.649 | 0.649 | 0.634 | 0.619 |

**3.2 Cross-Model Consistency**

The finding of preserved or improved performance after SAS exclusion was consistent across 8 of 9 algorithms on external validation AUC (Table S2). The only exception was LightGBM, where the difference was negligible (ΔAUC = −0.001). This cross-model consistency confirms that the result is not model-specific.

**Table S2.** External validation performance comparison across all 9 machine learning algorithms. 6-var = original model with SAS; 5-var = alternative model excluding SAS. Δ = 5-var minus 6-var. Positive Δ indicates improved performance after SAS exclusion.

| Algorithm | 6-var AUC | 5-var AUC | ΔAUC | 6-var F1 | 5-var F1 | ΔF1 |
| --- | --- | --- | --- | --- | --- | --- |
| LR | 0.910 | 0.901 | -0.010 | 0.71 | 0.68 | -0.03 |
| EN | 0.957 | 0.959 | +0.003 | 0.667 | 0.629 | −0.038 |
| XGBoost | 0.869 | 0.890 | +0.021 | 0.63 | 0.62 | -0.02 |
| RF | 0.883 | 0.903 | +0.020 | 0.62 | 0.63 | +0.02 |
| LightGBM | 0.845 | 0.877 | +0.032 | 0.60 | 0.62 | +0.02 |
| SVM | 0.862 | 0.908 | +0.046 | 0.611 | 0.649 | +0.038 |
| MLP | 0.770 | 0.622 | -0.149 | 0.55 | 0.00 | -0.55 |
| KNN | 0.831 | 0.835 | +0.004 | 0.62 | 0.59 | -0.03 |
| DT | 0.911 | 0.911 | +0.000 | 0.59 | 0.59 | +0.00 |

**3.3 SHAP Feature Importance (5-Variable Model)**

SHAP analysis of the 5-variable XGBoost model revealed the following feature importance ranking (mean |SHAP value|):

- Catheter-Related Complications: 1.435
- Peritonitis: 1.279
- SSRS Score: 0.699
- PD Vintage: 0.464
- Age: 0.380

Catheter-related complications and peritonitis remained the dominant predictors, consistent with the 6-variable model SHAP results, confirming that clinical complications—not psychological scale overlap—drive the model's discriminative power.

**4. Interpretation**

The sensitivity analysis yields three key insights:

1. No performance degradation: The 5-variable model (excluding SAS) achieved equal or superior performance across nearly all metrics on external validation (AUC +0.021, F1 +0.045). This directly refutes the concern that the original model's high performance was artifactually inflated by SAS-SDS overlap.
2. Cross-model consistency: 8 of 9 algorithms showed stable or improved AUC when SAS was excluded, confirming this finding is robust and not dependent on a specific modeling approach.
3. Clinical implication: The 5-variable model relies solely on clinical/demographic factors (age, peritonitis, catheter complications, social support, PD vintage), making it more practical for point-of-care screening in settings where concurrent anxiety assessment may not be available, and eliminating the concern about circular reasoning from shared psychological construct variance.

In conclusion, the model's discriminative utility demonstrably extends beyond mere redundancy with anxiety screening tools. The clinical complications (peritonitis, catheter-related complications) and social support provide independent, non-redundant predictive information for depression screening in PD patients.

**5. Figures**

**
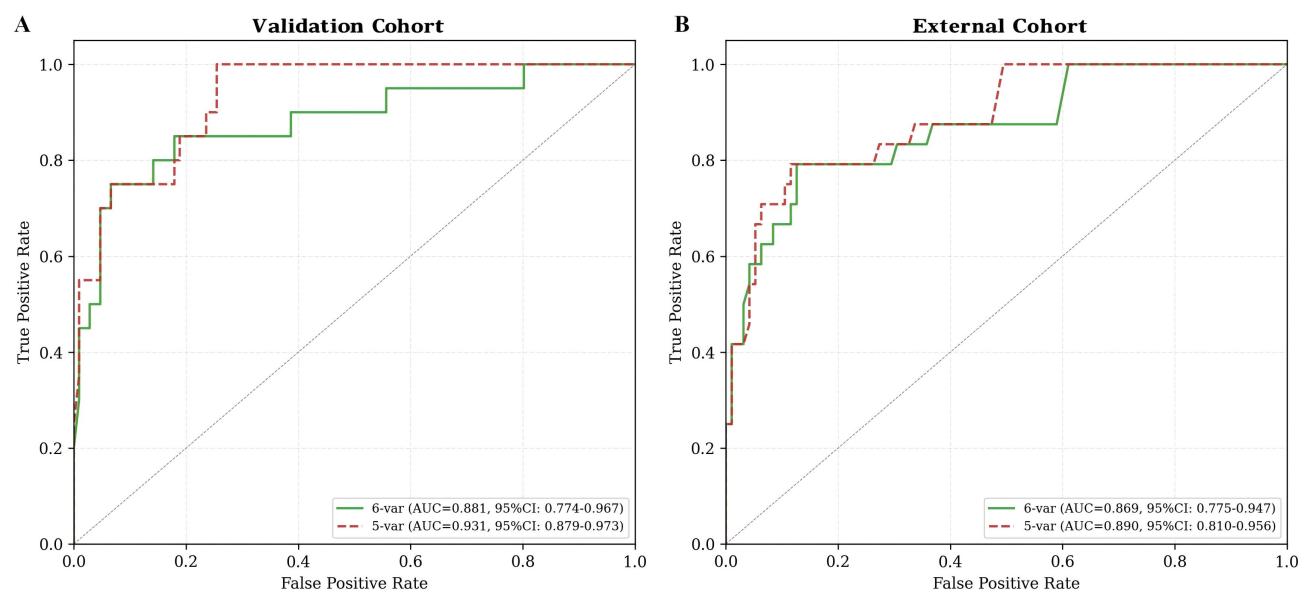
**

**Figure S1.** ROC curves comparing 6-variable (with SAS, solid blue line) and 5-variable (excluding SAS, dashed red line) XGBoost models. (A) Internal validation cohort (n=119); (B) External validation cohort (n=119). AUC values are shown for each model.


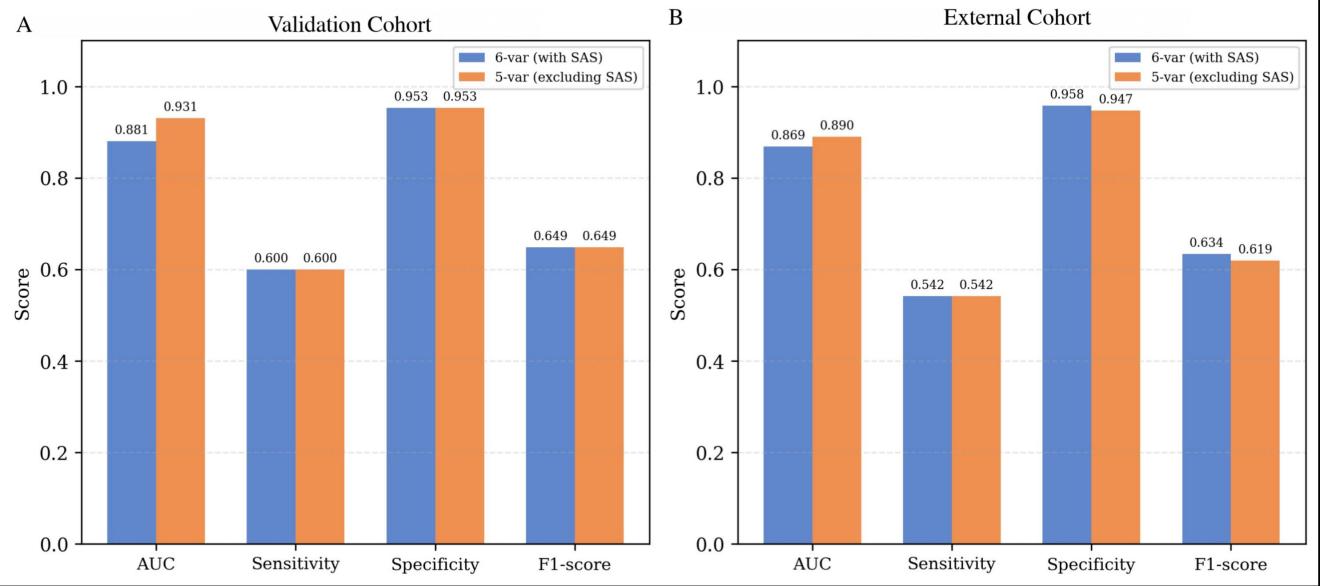


Figure S2. Performance metrics comparison of 6-variable (with SAS, blue bars) and 5-variable (excluding SAS, orange bars) XGBoost models. (A) Validation cohort (n=126); (B) External cohort (n=119). Metrics shown: AUC, Sensitivity, Specificity, and F1-score.


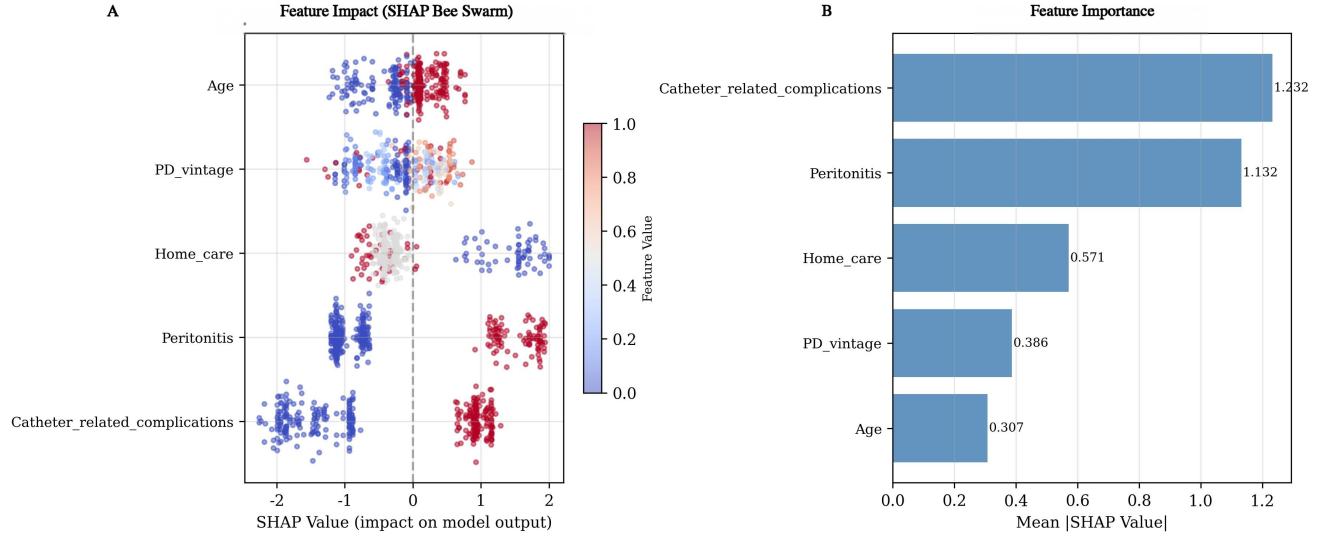


**Figure S3.** SHAP feature importance of the 5-variable XGBoost model (excluding SAS). (A) Beeswarm plot showing SHAP value distribution for each feature; (B) Bar plot of mean |SHAP| values. Catheter-related complications and peritonitis are the dominant predictors.
